# Revision of the genus *Niphta* (Diptera, Thaumaleidae) Theischinger of South America, with descriptions of nine new species and a new immature morphotype

**DOI:** 10.3897/zookeys.1063.71180

**Published:** 2021-10-19

**Authors:** Robert J. Pivar, Bradley J. Sinclair, John K. Moulton

**Affiliations:** 1 The University of Tennessee, Department of Entomology and Plant Pathology, 2505 E.J. Chapman Drive, 370 Plant Biotechnology Building, Knoxville, Tennessee, 37996, USA The University of Tennessee Knoxville United States of America; 2 Canadian National Collection of Insects and Canadian Food Inspection Agency, K.W. Neatby Building, C.E.F., 960 Carling Avenue, Ottawa, Ontario, Canada K1A 0C6 Canadian National Collection of Insects and Canadian Food Inspection Agency Ottawa Canada

**Keywords:** Andes, Chile, diversity, madicolous, midge, seepages

## Abstract

The *Niphta* Theischinger fauna of South America is revised to include 11 species, nine of which are described as new to science (*N.acus* Pivar, **sp. nov.**, *N.bifurcata* Pivar & Moulton, **sp. nov.**, *N.bispinosa* Pivar & Sinclair, **sp. nov.**, *N.brunnea* Pivar, **sp. nov.**, *N.courtneyi* Pivar, **sp. nov.**, *N.daniellae* Pivar, **sp. nov.**, *N.downesi* Pivar, **sp. nov.**, *N.eurydactyla* Pivar, **sp. nov.**, *N.mapuche* Pivar, **sp. nov.**). The genus *Niphta* is redefined, both previously described Chilean species are redescribed, *N.halteralis* (Edwards) and *N.nudipennis* (Edwards), and females are described or redescribed where possible. The first descriptions of the immature stages of South American *Niphta* are provided, which represent a new larval morphotype in Thaumaleidae, as larvae and pupae possess ventral adhesive structures. Furthermore, these larvae were collected from vegetation rather than rocky substrates. Illustrations and micrographs are provided for all species, and scanning electron microscopy images are included for select immatures. A key to species, distribution maps, and discussions regarding phylogenetic affinities and habitat are also included.

## Introduction

Thaumaleidae, or madicolous midges, consist of nearly 200 species classified in seven genera. They have no known medical or economic importance to humans, and are considerably less studied than their presumptive sister family, Simuliidae (black flies) (Moulton 2000; Bertone et al. 2008; Wiegmann et al. 2011; Borkent 2012; Kutty et al. 2018). Adults are commonly collected from rock-face seepages, margins of waterfalls and splash zones of cascading streams (Sinclair 2000; Pivar et al. 2018a, b; Pivar et al. 2020). Thaumaleid larvae are restricted to madicolous habitats, or substrates with a thin film of water flowing over them (Mackie 2004; Shimabukuro and Trivinho-Strixino 2018). Until this study, larvae were thought to be limited to rocky substrates.

South America has eight described species of Thaumaleidae in three genera prior to this study, known from only three countries: *Austrothaumalea* Tonnoir (five species, Chile and Argentina), *Neothaumalea* Pivar, Moulton and Sinclair (one species, Brazil) and *Niphta* Theischinger (two species, Chile). Edwards (1930) was the first to describe thaumaleids from South America, including three species of *Austrothaumalea* and two species of *Niphta*. Schmid (1970) later described an additional species of *Austrothaumalea*. The first record of the family from Brazil was published by Pivar et al. (2018b), where they described the new genus and species, *Neothaumaleaatlantica* Pivar & Pinho. Pivar et al. (2020) described an additional species of *Austrothaumalea*, synonymised *Oterere* McLellan with *Austrothaumalea* and provided a key to South American genera and species of *Austrothaumalea*. In addition to the described species above, Röder (1886) noted an undescribed species from the Ecuadorian Andes, though this record remains unverified by the authors despite attempts to locate the specimen.

The focus of this study is the South American *Niphta*, a small genus with two described species in Chile (Edwards 1930) and three in Australia (Theischinger 1986). The original descriptions by Edwards were brief and illustrations rudimentary, both severely lacking in detail. Discussions regarding relationships and keys were lacking, and immature stages unknown. Herein, *Niphta* is redefined, nine new species are described, Edwards’ species are redescribed and females are described or redescribed for all available species. Genitalic illustrations are included for all species and available sexes. Additionally, immature stages of South American *Niphta* are described for the first time. Distribution maps, keys to species (see Pivar et al. 2020 for key to genera), and discussions regarding phylogenetic affinities, faunal patterns and habitat are also provided.

## Materials and methods

Efforts were made to recollect fresh material from as near as possible to the perceived type localities. Adults were collected using an aerial net to sweep above the madicolous substrate and adjacent riparian vegetation. Sweeping for adults should be attempted first, if possible, to avoid having to sweep less accessible areas if they try to escape when searching for larvae. Immatures were collected by using forceps to pull them off the substrate, or by pouring water over the substrate and flushing immatures into a white pan (Sinclair and Saigusa 2002). In addition to examining rocks, vegetation (including leaves, stems, branches, etc.) in the splash zone should be carefully examined for larvae and pupae. This also includes inspecting any debris collected while sweep netting for adults, as it may contain immatures. Once collected, all life stages were placed directly into 75% non-denatured ethanol for morphological studies or 95% non-denatured ethanol for molecular studies.

Adult genitalia were cleared with hot 85% lactic acid. Representative adults and immatures were also cleared with the GeneJET Genomic DNA Purification Kit #K0722 (ThermoScientific, Waltham, MA) to maintain important membranous and lightly sclerotised structures, and to extract DNA for subsequent molecular study. The GeneJET lysate preparation protocol was followed and cleared voucher specimens were stored in 70% non-denatured ethanol. Positive identifications of females and immatures were made by comparing their DNA sequences to those of identified males. Specimens identified through molecular means are denoted by an asterisk (*) in the Type material and Additional material examined sections. Pinned specimens were dried using Brown’s (1993) hexamethyldisilazane (HMDS) method. Cleared terminalia were preserved in glycerine-filled microvials pinned beneath the specimen.

Specimens were viewed using a Meiji Techno RZ stereomicroscope mounted with a Progres Gryphax Naos 22-megapixel camera (Jenoptik, Jena, Germany) and aided by iSolution Lite x64 software (Focus Precision Industries, Victoria, MN, USA) to take light micrographs of pinned adults and immatures in alcohol. Image stacks were created using Helicon Focus 6.7.1 (HeliconSoft, Roseau Valley, Dominica). Cleared terminalia and larval head capsules in glycerine were viewed with an Olympus BH-2 compound microscope equipped with DIC and images were taken following the same methods as above. Line drawings were first traced from stacked micrograph images captured using the compound microscope, structures were re-evaluated by closely re-examining specimens (as stacking failed to clearly differentiate critical internal structures), then line drawings were inked and digitised for publication. The left gonocoxite and gonostylus were intentionally omitted for male *Niphtanudipennis* group species to allow for clearer visualisation of remaining genitalic characters.

Samples were prepared for scanning electron microscopy by transferring specimens from 95% non-denatured ethanol into a 12 mm × 30 μm microporous specimen capsule (Electron Microscopy Services, Hatfield, PA). Capsules were then subjected to the following HMDS dehydration series, each step lasting 20 minutes: 100% ethanol, 1:1 ethanol to HMDS, 1:75 ethanol to HMDS, then two steps of 100% HMDS. Dried specimens were mounted on carbon tape affixed to 45°/90° aluminium stubs and sputter-coated with gold for 10 sec at 20 μA in a SPI-Module Sputter Coater (West Chester, PA). Specimens were viewed with a Hitachi TM3030 electron microscope (Tokyo, Japan) at a voltage of 15 kV.

Terms used for adult structures follow Cumming and Wood (2017). Homology of the male terminalia follows Sinclair (1992). Terms used for larval and pupal structures follow those of Courtney et al. (2000) and Borkent (2012), respectively. The numbering system for larval head capsule setae and sensory pits follows Sinclair and Stuckenberg (1995). Distribution maps were created with SimpleMappr (Shorthouse 2010).

Specimens are deposited in the following repositories: Canadian National Collection of Insects, Ottawa, Canada (**CNC**); Instituto de Entomología, Universidad Metropolitana de Ciencias de la Educación, Santiago, Chile (**UMCE**); Museo Nacional de Historia Natural, Santiago, Chile (**MNNC**); National Museum of Natural History, Smithsonian Institution, Washington, D.C., USA (**USNM**); Robert J. Pivar, private collection, Ancaster, Ontario, Canada (**RJP**); University of Tennessee, Knoxville, Tennessee, USA (**UTK**).

Label data for primary types are presented exactly as they appear. Data are listed from the top downward on the staging pin, with data from each label enclosed in quotation marks; lines are delimited by a forward slash mark. Repository is given in parentheses and USNM database numbers are listed.

## Taxonomy

A key to adults of South American genera of Thaumaleidae is provided in Pivar et al. (2020).

### Genus Niphta

Taxon classificationAnimaliaDipteraThaumaleidae

Theischinger

B3593280-B791-59BE-9BD2-7F67C51A9C21

Niphta Theischinger, 1986: 314. Type-species: Niphtabickeli Theischinger (original designation).

#### Diagnosis.

*Niphta* is characterised as follows: presence of a distinct antealar ridge; proepisternal setae absent; microtrichia of R_1_(+R_2+3_) confined to base near humeral crossvein; R_2+3_ crossvein situated closer to apex of R_1_(+R_2+3_) than to origin of R_4+5_; R_1_ and R_1_(+R_2+3_) with three weakenings or depigmented gaps; R_4+5_ often with arch not strongly produced; basal spur of CuA absent; gonocoxites broad, not much longer than wide; hypandrium absent; gonocoxal plate extended posterodorsally forming a medial process; parameres fused medially, emerging from gonocoxal plate complex.

#### Redescription.

*Adult*. Eye bridge broad, comprising more than five facets. Scutum clothed in both short and long setae; scutellum with row of long, black marginal setae. Supra-alar region produced into distinct antealar ridge (Fig. 9H); ridge with setae. Proepisternal setae absent. Wing tip narrowly rounded; membrane lacking macrotrichia; C with sparse macrotrichia, more so on remaining wing margin; Sc incomplete; microtrichia of R_1_(+R_2+3_) confined to base near humeral crossvein; R_2+3_ crossvein situated closer to origin of R_4+5_ than to apex of R_1_(+R_2+3_); R_1_ and R_1_(+R_2+3_) with three weakenings or depigmented gaps; R_4+5_ often with arch not strongly produced; R_4+5_ and M_1_ running parallel toward margin; M_1_ straight; M_2_ with gentle bend in apical third; M_4_ with slight bend; CuA angulate near base; basal spur of CuA absent. *Male Terminalia*: Hypandrium absent. Gonocoxites broad, not much longer than wide; gonocoxal plate extended posterodorsally, forming medial process, acting as aedeagal guide; parameres fused medially, emerging from gonocoxal plate complex.

#### Distribution.

Chile and Australia.

#### Species groups.

Prior to this study, only five described species of *Niphta* were known from all regions and few phylogenetic affinities had been discussed. Theischinger (1986) suggested that the Australian *N.farecta* Theischinger was more closely allied to the Chilean *N.nudipennis* (Edwards) than the other Australian species. With the additional nine species described herein, it is now possible to better assess relationships based upon morphology. Species groups are proposed below for the genus *Niphta*.

The *N.bickeli* group: This group is characterised by the following features: broad gonocoxites extending to the posterior epandrial margin and lacking projections; long gonostyli; parameres fused medially, then separating into two arms that do not project anteriorly; a pair of apodemes from base of parameres extend to posterior margin of epandrium, on either side of the anus; cerci inconspicuous, thinly sclerotised and unpigmented; females lack projection at base of hypogynial valves. This group is greyish black in colouration. Additionally, immatures of *N.collessi* Theischinger lack ventral adhesive structures and protuberances on the larval head capsule (Sinclair 2000); these are tentatively seen as important characters uniting this species group until further Australian immatures are discovered and accurately associated with the adults (see discussion below comparing *Niphta* immatures). The *N.bickeli* group is found in Australia and includes the following species: *N.bickeli* Theischinger, *N.collessi*, and *N.farecta*.

The *N.halteralis* group: This group is characterised by the following features: gonocoxites extending to midpoint of epandrium, lacking pointed projections; short and narrow gonostyli; parameres fused throughout; prominent cerci projecting anteriorly, extending well beyond posterior epandrial margin; females lack projection at base of hypogynial valves. This group is darkly coloured, mostly black and grey. Immature stages have ventral adhesive structures, are collected from rocky substrates and larval head capsules have many protuberances. The *N.halteralis* group is known from Chile and includes the following species: *N.acus* sp. nov., *N.downesi* sp. nov., *N.halteralis* (Edwards), and *N.mapuche* sp. nov.

The *N.nudipennis* group: This group is characterised by the following features: gonocoxites extending, at most, to midpoint of epandrium, and bearing pointed projections; broad, short gonostyli cheliform or finger-like; parameres fused medially. separated into two arms that project anteriorly and may be flexed or extended; cerci small, projecting anteroventrally; females possess distinct blunt or pointed projection at base of hypogynial valves; sternite 8 highly modified (genital fork and lateral arms); Female sternite 9 greatly expanded and heavily sclerotised, presumably reflecting the highly modified male genitalia. This group tends to be lighter in colouration. Immature stages have ventral adhesive structure, are collected from vegetation in splash zones, and larval head capsules have many protuberances. The *N.nudipennis* group is known from Chile and includes: *N.bifurcata* sp. nov., *N.bispinosa* sp. nov., *N.brunnea* sp. nov., *N.courtneyi* sp. nov., *N.daniellae* sp. nov., *N.eurydactyla* sp. nov. and *N.nudipennis*.

### Key to adult males of South American species of Niphta^*^

**Table:** 

1	Gonocoxite subquadrate or conical, with posteromedial projection broad, rounded, projecting posteriorly (Fig. 2). Parameres fused medially to apex (Figs 2, 3). Body typically dark in colour (Fig. 4)	**2** (***N.halteralis* group**)
–	Gonocoxite oblong, with posteromedial projection narrow, pointed, projecting medially (Figs 5, 6). Parameres fused medially then separating into two raptorial-like apical arms (Figs 7, 8). Body typically light in colour (Figs 9, 10)	**5** (***N.nudipennis* group**)
2	Paramere with hooked apex. Gonostylus straight (Figs 2C, 3C)	***N.halteralis* (Edwards)**
–	Paramere without hooked apex. Gonostylus arched outwards	**3**
3	Gonostylus bifurcate apically. Paramere with abrupt, strongly tapered, off-centred needle-like apex (Figs 2A, 3A)	***N.acus* Pivar, sp. nov.**
–	Gonostylus tapered to single apex. Paramere evenly tapered throughout	**4**
4	Paramere, in lateral view, divided into two filaments; dorsal filament, at most, nearly reaching posterior margin of epandrium; ventral filament not extended beyond apex of gonostylus, not easily visible (Figs 2D, 3D)	***N.mapuche* Pivar, sp. nov.**
–	Paramere, in lateral view, divided into three filaments; dorsal filament extended beyond posterior margin of epandrium; paired ventral filaments extended beyond apex of gonostylus, easily visible (Figs 2B, 3B)	***N.downesi* Pivar, sp. nov.**
5	Gonostyli cheliform or bearing a finger-like projection (Figs 5B, C, 6)	**6**
–	Gonostyli not cheliform, without projections; broad at base, tapered to pointed apex (Figs 5A, 7A)	***N.daniellae* Pivar, sp. nov.**
6	Gonostyli with finger-like projection (Fig. 5B, C)	**7**
–	Gonostyli cheliform, resembling crab claw (Fig. 6)	**8**
7	Gonostyli with projection broad, tapered slightly at apex, without bend (Figs 5B, 7B)	***N.eurydactyla* Pivar, sp. nov.**
–	Gonostyli with projection narrow throughout, bent at midpoint (Figs 5C, D, 7C, D)	***N.nudipennis* (Edwards)**
8	Gonostyli with posterior apex bifurcate (Fig. 6A, B)	**9**
–	Gonostyli with posterior apex bearing single apex (Fig. 6C, D)	**10**
9	Gonocoxites with two projections, anterior one bifurcate (Figs 6A, 8A). Body brown to dark brown in colour (Figs 9E, 10E)	***N.bifurcata* Pivar & Moulton, sp. nov.**
–	Gonocoxites with three separate projections (Figs 6B, 8B). Body yellow in colour (Figs 9B, 10B)	***N.courtneyi* Pivar , sp. nov.**
10	Gonocoxites with two projections; anterior one long, bifurcate; posterior one small, tooth-like (Figs 6C, 8C). Body yellowish brown in colour (Figs 9G, 10G)	***N.bispinosa* Pivar & Sinclair, sp. nov.**
–	Gonocoxites with three projections, two anterior (one small and inconspicuous at base of large one), one posterior; posterior projection slender, about as long as larger anterior projection (Figs 6D, 8D). Body dark brown in colour (Figs 9A, 10A)	***N.brunnea* Pivar, sp. nov.**

### Key to adult female species groups of South American *Niphta*

**Table:** 

1	Sternite 8 with distinct projection between hypogynial valves (Figs 11, 12)	***N.nudipennis* group**
–	Sternite 8 without distinct projection between hypogynial valves (Fig. 13)	***N.halteralis* group**

### Species diagnoses and descriptions of South American *Niphta*

**The *N.halteralis* group**

### Niphta acus

Taxon classificationAnimaliaDipteraThaumaleidae

Pivar sp. nov.

B16C7280-B117-562A-924B-36F7203A17ED

http://zoobank.org/4E68FECD-24B7-4E34-9F20-0F8FCC80EDBD

Figs 2A , 3A , 4A , 13A, B , 14C , 15C , 16C , 17C , 18C , 19C , 20A, B , 25 , 27C

#### Type material.

***Holotype*:** ♂, glued to point with abdomen in glycerine microvial pinned beneath, labelled: “Chile: Region VIII (Bío Bío)/ Rte. Q-61, Estero Agua/ Blanca, 8.xii.2016/ 37°46'30.8"S 71°42'03.9"W/ elev. 552 m, vegetation near/ splash zones, J.K. Moulton &/ R.J. Pivar”; “HOLOTYPE/ *Niphta*/ *acus*/ Pivar [red label]” (CNC). ***Allotype***: ♀, same label data as holotype (CNC). ***Paratypes***: Chile: Region RM (Santiago): Quebrada el Cinco Mil, 17.xii.2016, 33°31'30.4"S 70°13'52.6"W, elev. 1308 m, creek, J.K. Moulton & R.J. Pivar (3♀*); Region V (Valparaíso): Rte. 60, 18.xii.2016, 32°54'31.3"S 70°18'21.5"W, elev. 1423 m, creek, J.K. Moulton & R.J. Pivar (2♂); Region VIII (Bío Bío): Rte. N-55, 16.xii.2016, 36°55'02.7"S 71°25'49.6"W, elev. 1449 m, roadside seep, J.K. Moulton & R.J. Pivar (25♂); same label data as previous except, collected from rockface (8 larvae*, 5 pupae*, 8 pupal exuviae); Rte. Q-61, Estero Agua Blanca, 8.xii.2016, 37°46'30.8"S 71°42'03.9"W, elev. 552 m, vegetation near splash zones, J.K. Moulton & R.J. Pivar (41♂, 10♀*, 1 larva*); Rte. Q-61, 8.xii.2016, 37°48'34.7"S 71°40'30.0"W, elev. 390 m, roadside seep, J.K. Moulton & R.J. Pivar (2♂, 1♀*).

**Figure 1.**
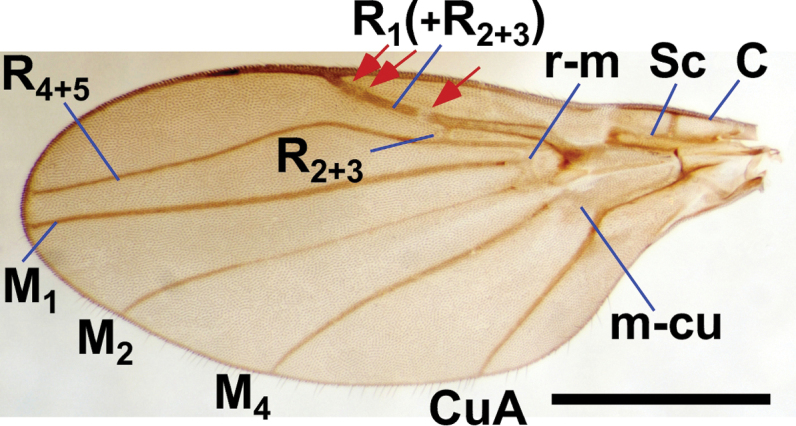
Left wing of *Niphtanudipennis* with arrows indicating three depigmented gaps. Abbreviations: C, costa; CuA, anterior branch of cubital vein; M, medial veins; m-cu, medial-cubital crossvein; R, radial veins; r-m, radial-medial crossvein; Sc, subcosta. Scale bar: 1.0 mm.

**Figure 2.**
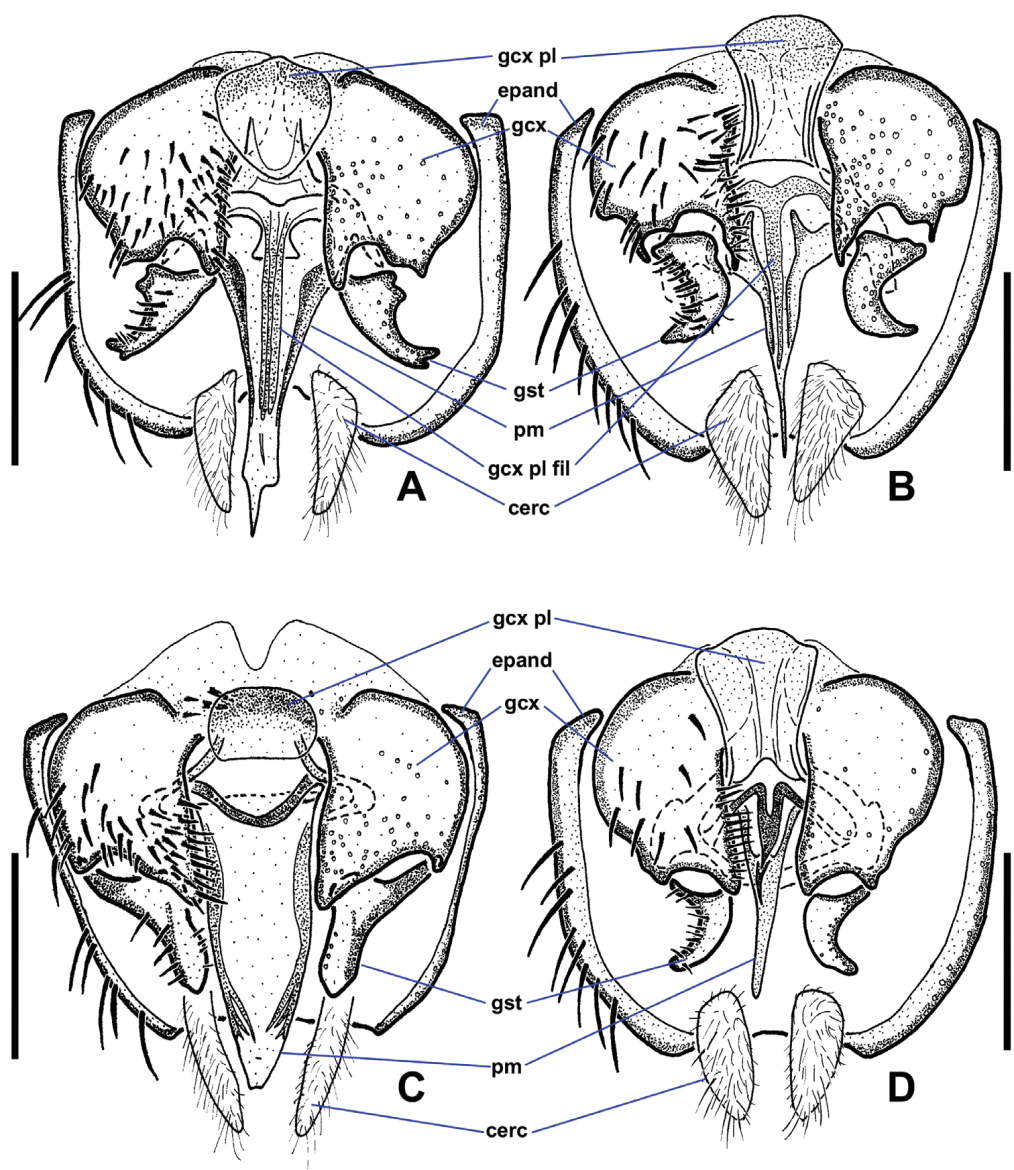
Ventral views of male *Niphtahalteralis* group terminalia **A***N.acus* sp. nov. **B***N.downesi* sp. nov. **C***N.halteralis***D***N.mapuche* sp. nov. Abbreviations: cerc, cercus; epand, epandrium; gcx, gonocoxite; gcx pl, gonocoxal plate; gcx pl fl, gonocoxal plate filament; gst, gonostylus; pm, paramere. Scale bars : 0.1 mm.

**Figure 3.**
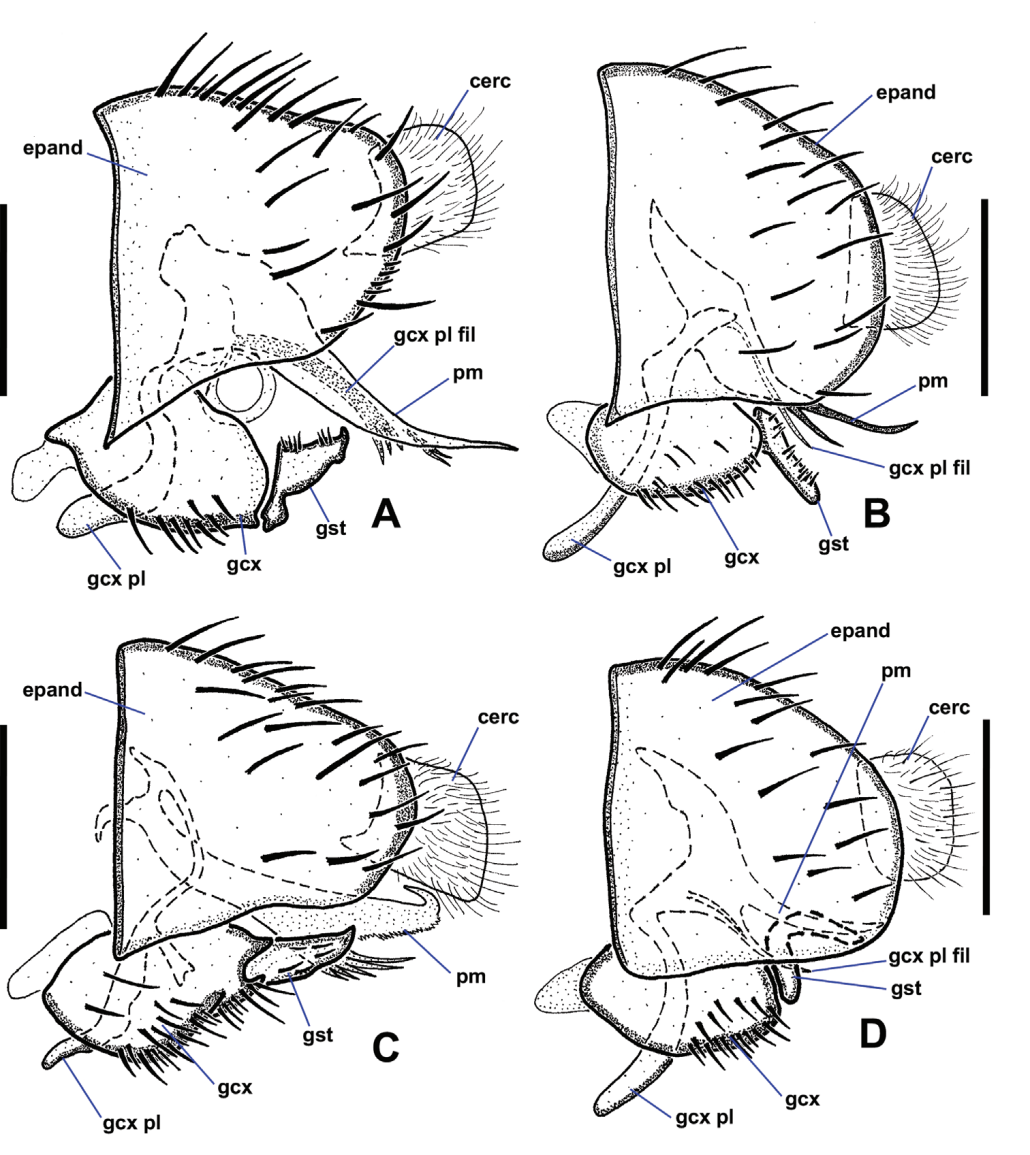
Lateral views of male *Niphtahalteralis* group terminalia **A***N.acus* sp. nov. **B***N.downesi* sp. nov. **C***N.halteralis***D***N.mapuche* sp. nov. Abbreviations: cerc, cercus; epand, epandrium; gcx, gonocoxite; gcx pl, gonocoxal plate; gcx pl fl, gonocoxal plate filament; gst, gonostylus; pm, paramere. Scale bars: 0.1 mm.

#### Recognition.

*Niphtaacus* is recognised by the bifurcated apex of the gonostylus and the strongly apically tapered parameres, giving the appearance of a needle-like tip.

#### Description.

**Male.***n* = 71.

*Length* 1.6–2.5 mm.

**Figure 4.**
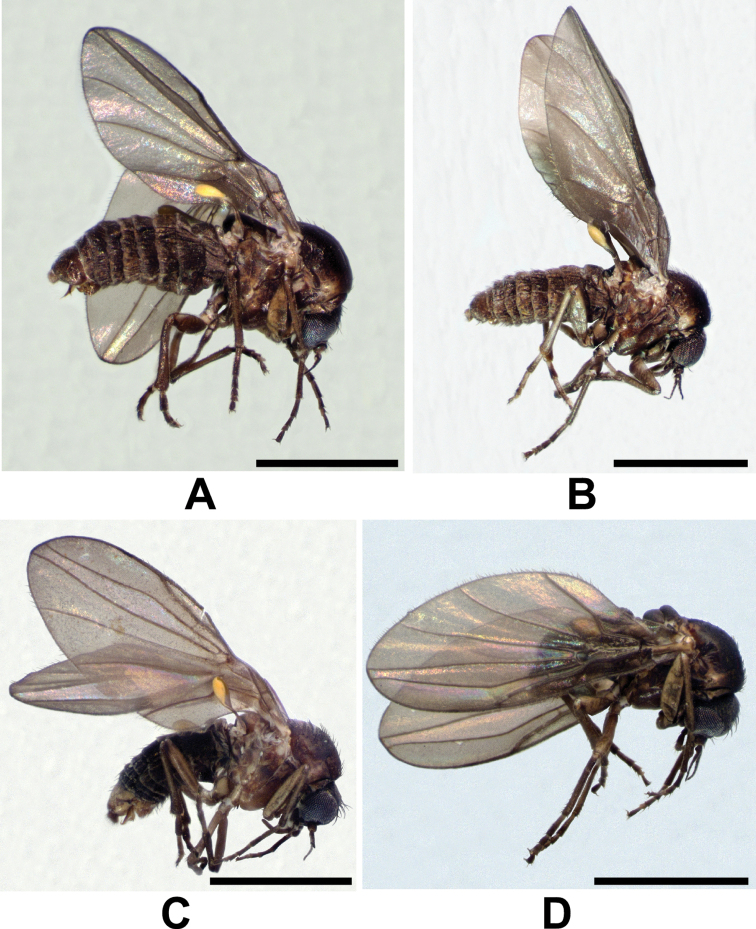
Adult male lateral habitus micrographs of the *Niphtahalteralis* group **A***N.acus* sp. nov. **B***N.downesi* sp. nov. **C***N.halteralis***D***N.mapuche* sp. nov. Scale bars: 1.0 mm.

*Colouration* (Fig. 4A). Head dull, blackish brown; pronotum and postpronotum dark brown; postpronotal lobe brown with dark brown markings, light brown around anterior spiracle; prescutum, mesoscutum and pleura shiny, blackish brown; scutellum and mediotergite shiny, blackish brown; katepisternum dark brown with blackish brown markings, remaining pteropleuron mainly blackish brown with dispersed markings of brown to light brown; base of halter black, knob creamy yellow; legs greyish brown, apex of tarsi darker; abdomen blackish brown; terminalia concolourous with abdomen.

*Head*. Eyes above antennae broadly joined, with small triangular frons visible above antennae; frons with three to five strong setae. Flagellomeres 1–3 subquadrate, with flagellomere 1 expanded, 3 × as wide as next segment, equal to lengths of 2 and 3 combined; flagellomeres 4–10 cylindrical, becoming progressively thinner and elongate. Vertex with black setae of uniform length, with longer, black orbital setae.

*Thorax*. Mesoscutum with prominent antealar ridge, bearing thee pronounced setae. Scutum clothed dorsally in short, black setulae; notopleural, supra-alar and postsutural setae long, black. Pteropleuron bare. All legs with tarsi simple.

*Wing*. Wing length: 1.8–2.5 mm. Dark, infuscate throughout, apex somewhat narrowed; C fringed in small setulae, with a few microtrichia scattered throughout; posterior wing margin with closely spaced fringe of microtrichia; Sc incomplete; R_1_ and R_1_(+R_2+3_) with three weakenings or depigmented gaps, first slightly beyond R_2+3_, second and third closely approximated, near C; microtrichia of R_1_(+R_2+3_) confined to base near humeral crossvein, remaining veins bare; R flexed into cell br; R_2+3_ distinct, situated in basal third of R_1_(+R_2+3_); bend in R_4+5_ gentle; R_4+5_ and M_1_ running parallel toward margin; M_1_ straight; M_2_ with gentle bend in apical third; M_4_ with slight bend.

*Abdomen*. Abdominal sternite 1 narrow, spectacle-shaped; sternite 2 reduced to slender median sclerite, a few setae restricted to laterad on posterior third and medially beneath sclerite; sternites 3–7 rectangular, lacking distinct sclerites, setae restricted to posterior two-thirds; sternite 8 strongly reduced, anterior margin well sclerotised, arched slightly into preceding segment, lacking setae.

*Terminalia* (Figs 2A, 3A). Epandrium quadrate in ventral view, posterior margin rounded with large, medial indentation; long, extending well beyond gonostyli; without lobes or projections. Gonocoxites conical, one-third longer than wide, anterior margin rounded, expanded dorsally behind gonocoxal plate, nearly meeting medially, extended anteriorly toward sternite 8; posterior inner margin produced into rounded projection, outer margin without notch; inner margin densely setose. Gonostylus short, three-quarters length of cercus, strongly curved laterally throughout; widest at base, tapered toward bifurcated apex; outer margin bearing laterally directed setae. Parameres fused at gonocoxal apodeme, widest at point of fusion; extended beyond cerci; forming canal-like structure, flattened apically, tapered to off-centre sharp point (occasionally specimens with broken apex). Gonocoxal plate well sclerotised; tongue-like plate extended anteroventrally; hollow medially, pitcher plant-like; gonocoxal apodeme with secondary structure comprising three arms fused medially, running along interior of paramere canal, flaring into three or four filaments, projected ventrally at apex, with pair of lateral flanges near point of fusion. Cercus large, prominent; subquadrate; projected posteriorly; situated within epandrial indentation.

**Female.***n* = 15.

Similar to male except as follows: *Terminalia* (Fig. 13A, B). Hypogynial valve not projecting beyond tergite 9; posterior margin deeply emarginated in ventral view, nearly dividing sternite in half, forming two subtriangular lobes; lobes densely setose. Tergite 9 subtriangular in lateral view, 3 × as wide as tergite 8, lacking lateral projections; posterior margin heavily sclerotised at base of cerci. Sternite 9 (genital fork) slender, Y-shaped anteriorly and posteriorly; lateral arms extended slightly beyond hypogynial valve, divergent toward apex. Hypoproct sclerotised, narrow. Cercus rounded, projected posteroventrally; bearing numerous setae.

**Figure 5.**
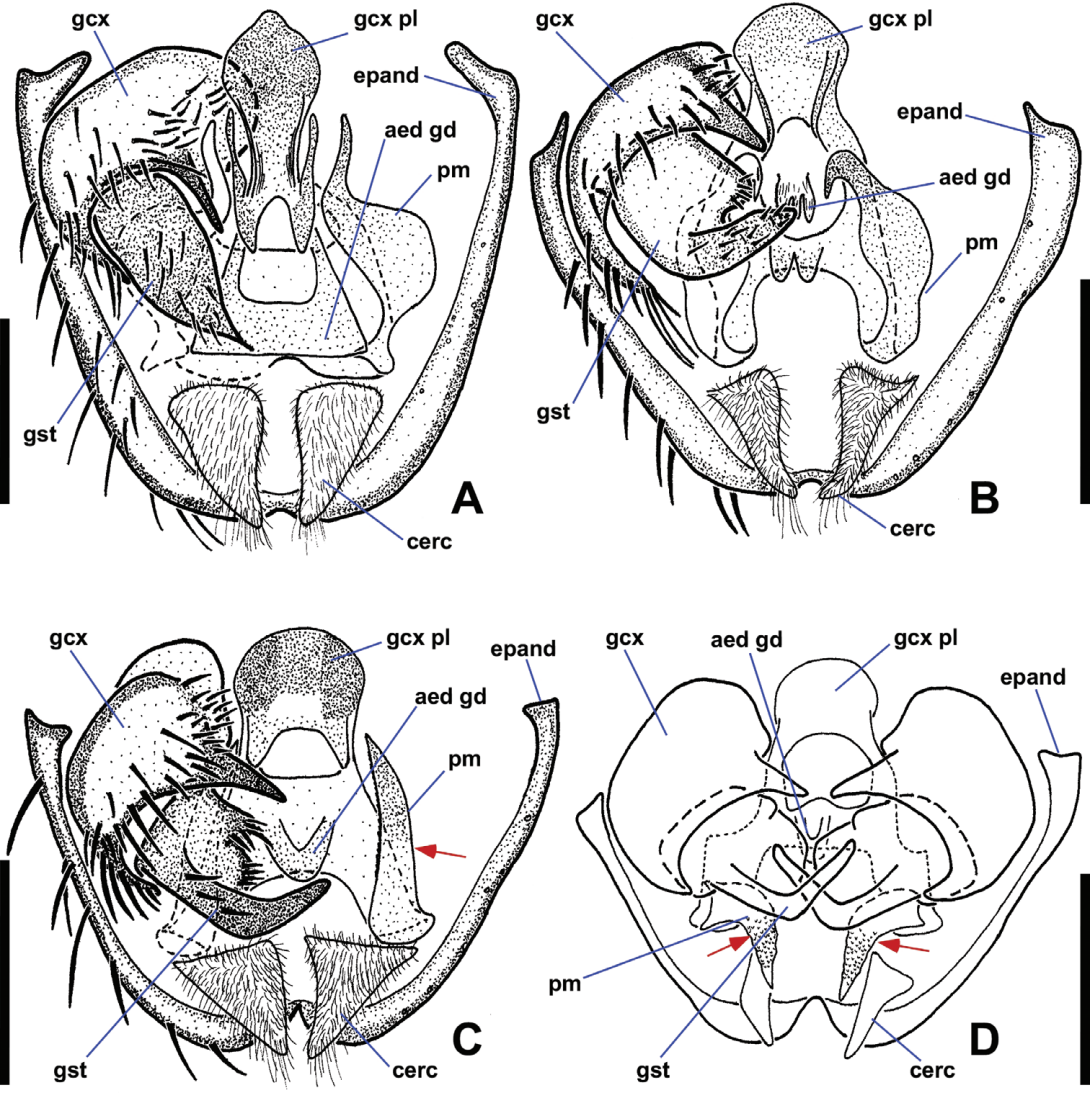
Ventral views of male *Niphtanudipennis* group terminalia **A***N.daniellae* sp. nov. **B***N.eurydactyla* sp. nov. **C***N.nudipennis* with parameres retracted **D***N.nudipennis* with parameres extended. Arrows indicate range of motion of parameres. Abbreviations: aed gd, aedeagal guide; cerc, cercus; epand, epandrium; gcx, gonocoxite; gcx pl, gonocoxal plate; gst, gonostylus; pm, paramere. Scale bars: 0.1 mm.

**Pupa.***n* = 8 (Figs 14C, 15C, 16C).

*Length* 3.0–4.0 mm.

*Colouration*. Brown; with black spot above eye in developing adult.

*Head*. Maxillary sheath short, posteromedially directed; gently tapered toward truncate apex; apices of palpi separated medially. Three short, slender setae above black spot over eye.

**Figure 6.**
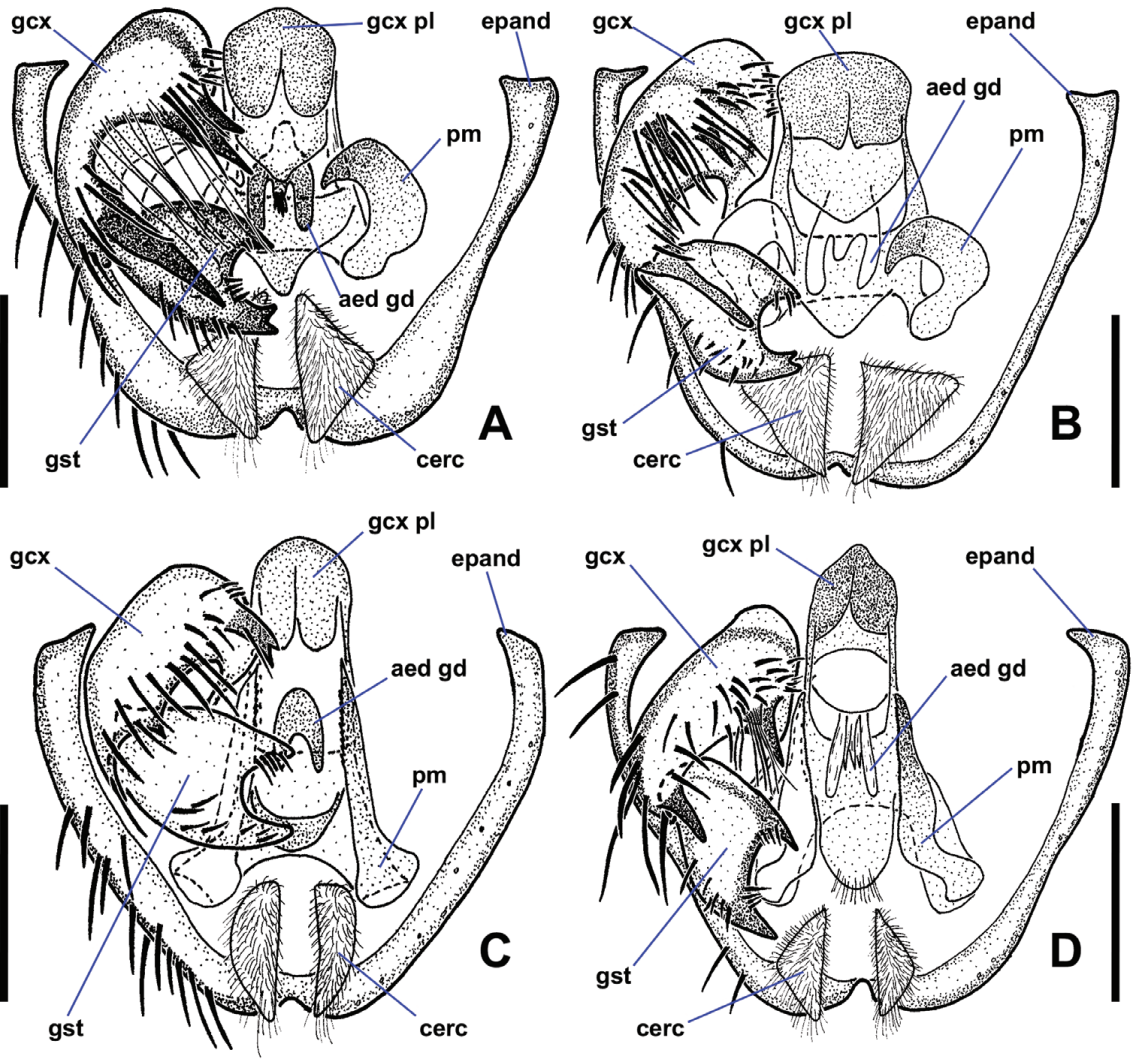
Ventral views of male *Niphtanudipennis* group terminalia **A***N.bifurcata* sp. nov. **B***N.courtneyi* sp. nov. **C***N.bispinosa* sp. nov. **D***N.brunnea* sp. nov. Abbreviations: aed gd, aedeagal guide; cerc, cercus; epand, epandrium; gcx, gonocoxite; gcx pl, gonocoxal plate; gst, gonostylus; pm, paramere. Scale bars: 0.1 mm.

*Thorax*. Width nearly subequal to abdomen at widest point. Foreleg sheath projecting straight, slightly longer than wing sheaths, reaching posterior margin of sternite 2; anterior half of midleg visible anterior to wing sheath, then hidden behind foreleg, slightly shorter than foreleg; hind leg concealed behind wing sheath, only apex visible between apex of foreleg and wing sheath, shorter than foreleg. Wing sheaths not reaching posterior margin of abdominal sternite 2; large tubercle at base bearing pair of short, slender setae. Respiratory organ short and squat, much shorter than maxillary sheath, broadest subapically; bulbous; spiracular openings encircling apex; stalk wide, emerging from small tubercle. Tubercle situated posterodorsally to respiratory organ, rounded, projected slightly laterally; apex nearly touching or touching respiratory organ. Tubercle situated posterolaterally to respiratory organ crenulate, projected slightly anteriorly. Ridge located anteroventrally to respiratory organ with single, thin midlateral seta; mesothorax with group of four short, slender dorsocentral setae near ridge; single seta on humeral lobe.

**Figure 7.**
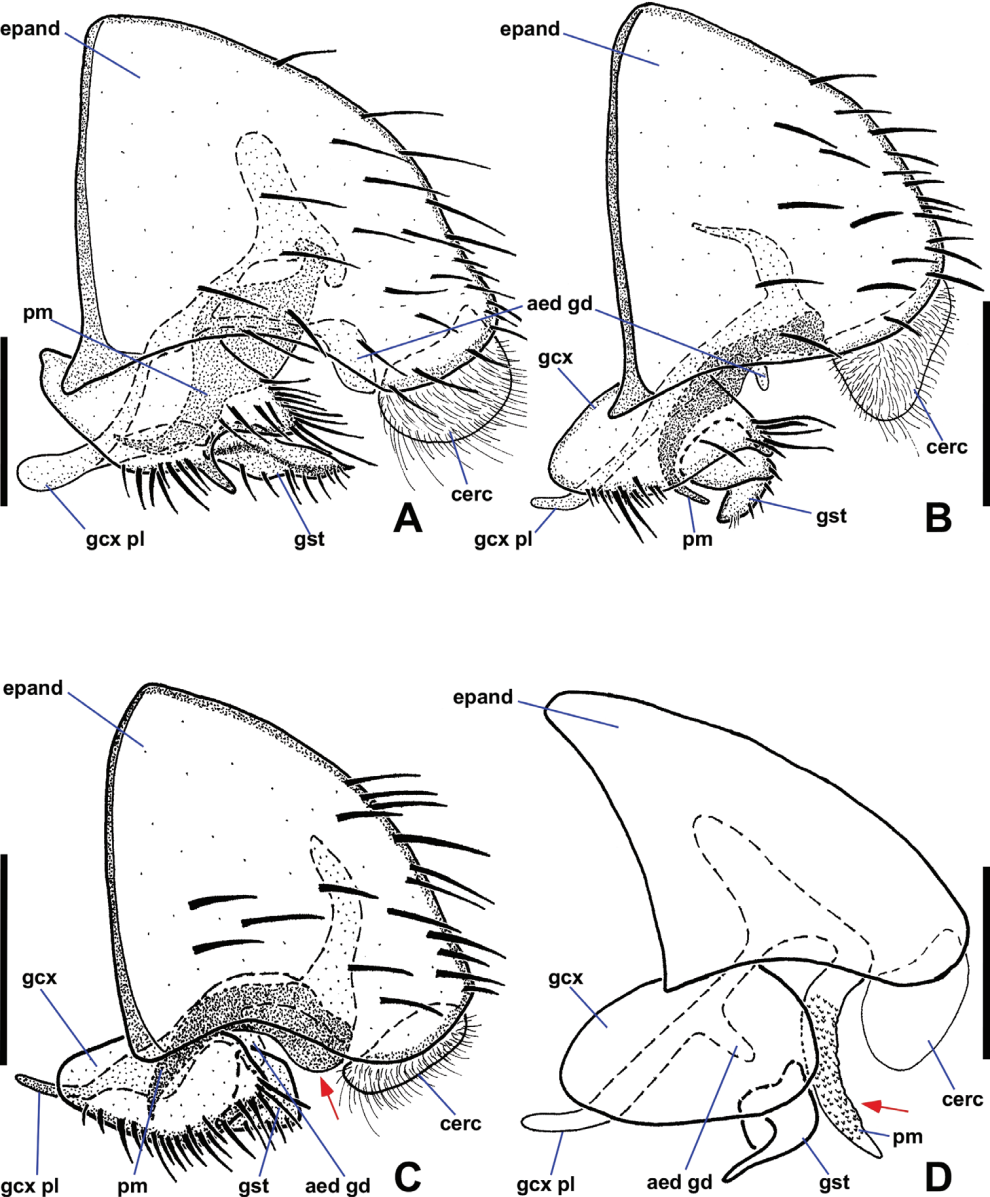
Lateral views of male *Niphtanudipennis* group terminalia **A***N.daniellae* sp. nov. **B***N.eurydactyla* sp. nov. **C***N.nudipennis* with parameres retracted **D***N.nudipennis* with parameres extended. Arrows indicate range of motion of parameres. Abbreviations: aed gd, aedeagal guide; cerc, cercus; epand, epandrium; gcx, gonocoxite; gcx pl, gonocoxal plate; gst, gonostylus; pm, paramere. Scale bars: 0.1 mm.

**Figure 8.**
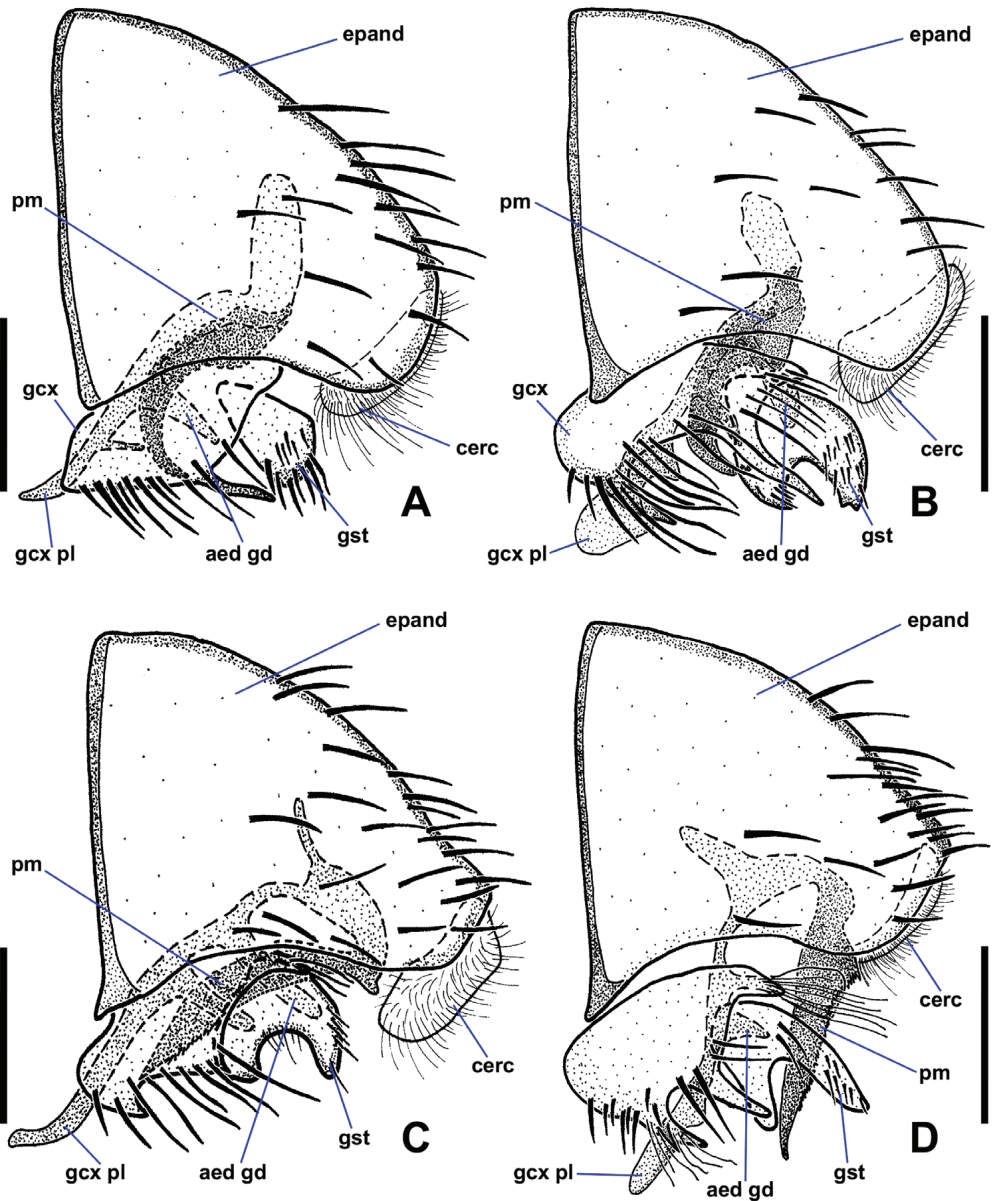
Lateral views of male *Niphtanudipennis* group terminalia **A***N.bifurcata* sp. nov. **B***N.courtneyi* sp. nov. **C***N.bispinosa* sp. nov. **D***N.brunnea* sp. nov. Abbreviations: aed gd, aedeagal guide; cerc, cercus; epand, epandrium; gcx, gonocoxite; gcx pl, gonocoxal plate; gst, gonostylus; pm, paramere. Scale bars: 0.1 mm.

*Abdomen*. Subcylindrical, strongly tapered at caudal segment. Spiracles weakly developed, not projected or distinctly visible. Tergites 1–8 rectangular, without ridges; bearing pair of slender lateral setae above lateral margins and pair of slender dorsolateral setae. Tergite 9 rounded, posterior with dorsolateral ridges bearing pair of lateral setae and hind margin emarginated; projection directed posteriorly in lateral view. Sternites 3–8 rectangular, without row of faint setulae along anterior margin; lateral margins crenulate, bearing a few thin, short setae. Sternites 3 and 4 bearing pair of small lateral adhesive structures, sternite 5 bearing pair of large lateral adhesive structures on lateral margin. Caudal sternite subquadrate, with pair of posteriorly projected medial lobes; posterior margin with pair of medial ridges, curved dorsally forming small, dorsally projected tubercle in lateral view; without distinct caudal hooks.

**Larva.***n* = 8 (Figs 17C, 18C, 19C, 20A, B).

*Length of final instar* 6.2–6.7 mm.

*Colouration*. Head capsule usually black or dark brown, sometimes black with light brown markings. Body mottled with various shades of grey and brown.

*Head capsule* (Fig. 20A, B). Two large, circular eye spots, elevated on tubercle; antenna on largest tubercle, with three finger-like processes; with five pairs of tubercles outside of ecdysial lines (not including antennal and ocular tubercle), two outermost tubercles bifurcate; single tubercle between ecdysial line, bi- or trifurcate; 15 pairs of unbranched setae; five pairs of sensory pits (13, 14, 18, 20, 21), sensory pit 13 above antennal tubercle.

*Thorax*. Prothorax with pair of anterodorsolateral protuberances bare; anterolateral protuberances with one long and two short setae; spiracular protuberance bearing two protuberances, inner protuberance with pair of setae, outer with single seta; pair of midlateral setae below anterolateral protuberance; three closely approximated setae near base of prothoracic leg (Keilin’s organ). Mesothorax and metathorax with pair of dorsolateral protuberances bearing pair of closely approximated setae, one thickened, one slender; mesothorax with additional seta beneath protuberance; lateral protuberance on both segments bearing four setae; one long seta slightly ventral to lateral protuberance; three mid-ventrolateral setae directed ventrally. Prothorax bearing proleg, posterior half with rectangular adhesive structure; meso- and metathoracic sternites with rectangular adhesive structures.

*Abdomen*. Sternites 1–7 modified into circular, suction cup-like adhesive structures; sternite 8 with quadrate adhesive structure, extending over sternite 9; sternite 9 smooth, bearing anal proleg. Tergites 1–7 with single anterolateral protuberance on each side with single seta, and pair of posterodorsolateral protuberances, each bearing two closely approximated short, thin setae; lateral adhesive structure swelling bearing four setae, two lateral, two basal; additional single seta located anterior to lateral swelling. Segment 8 with dorsolateral protuberance on either side of posterior spiracular plate, each bearing pair of small setae; lateral protuberance with three setae; single short ventrolateral seta; ventral surface bearing pair of setae. Posterior spiracular plate with sclerite encircling procerci; procercus shorter than length of spiracular plate, bearing four setae, two thick, two slender; without cone-like protuberance on either side of procerci. Terminal segment with anterior dorsal protuberance bare; pair of posterior lateral protuberances with pair of setae; five lateral setae; two pairs of long setae on posterior margin, above pair of anal papillae; ventral sternite bearing single pair of setae.

#### Additional material examined.

Known only from the type series.

#### Distribution.

Known from the south-central Andes of Chile (Fig. 24A); this species includes the northern-most record for *Niphta* in South America.

#### Etymology.

The species name is from the Latin *acu* (needle, pin) in allusion to the needle-like tip of the paramere.

#### Bionomics.

This species was collected at higher elevations than any other species in this publication. Larvae and pupae have ventral adhesive structures and were collected only from rocky substrates (Fig. 25). All immatures were collected from the margin of waterfalls, where water flow was quite slow.

### Niphta downesi

Taxon classificationAnimaliaDipteraThaumaleidae

Pivar sp. nov.

623605C3-6690-5BF7-9A46-F3E509F742F8

http://zoobank.org/11210E93-FC16-4315-BD7A-8974A93E782C

Figs 2B , 3B , 4B , 24A , 27A

#### Type material.

***Holotype*:** ♂, glued to point with abdomen in glycerine microvial pinned beneath, labelled: “Chile: Region XIV (Los Ríos)/ Rte. T-85, 13.xii.2016/ 40°19'58.6"S 72°16'56.1"W/ elev. 95 m, roadcut seep, J.K./ Moulton & R.J. Pivar”; “HOLOTYPE/ *Niphta*/ *downesi*/ Pivar [red label]” (CNC). ***Paratypes***: Chile: Region X (Los Lagos): Ensenada, nr. Baños de Petrohué, 12.i.1985, J.A. Downes (1♂, CNC); Region XIV (Los Ríos): same data as holotype (1♂); Rte. T-85, 13.xii.2016, 40°19'58.7"S 72°16'54.8"W, elev. 145 m, foliage around waterfalls, J.K. Moulton & R.J. Pivar (12 ♂).

#### Recognition.

This species is recognised by both filaments of the paramere and the aedeagal guide being easily visible in lateral view, giving it a tri-filamentous appearance. Also, the bend in the gonostylus is slightly stronger than that of *N.mapuche*.

#### Description.

The description of *N.downesi* differs from that of *N.acus* in the following regards:

**Male.***n* = 3.

*Length* 1.9–2.4 mm.

*Colouration* (Fig. 4B). Head dull, black; pronotum and postpronotum black; postpronotal lobe, prescutum and mesoscutum shiny, blackish brown, lateral face of postpronotal lobe brown, creamy around anterior spiracle; scutellum blackish brown dorsally, light brown ventrally; mediotergite shiny, blackish brown; pteropleuron mainly brown with dispersed markings of black and dark brown; base of halter blackish, knob creamy yellow; abdomen blackish brown.

*Head*. Frons with two strong setae. Flagellomeres 1–3 subquadrate, expanded, 1.5 × as wide as next segment, as long as 2 and 3 combined.

*Thorax*. Antealar ridge bearing single pronounced, medial seta flanked by two smaller setae.

*Wing*. Wing length: 2.2–2.4 mm. C and posterior wing margin with fringe of microtrichia.

*Abdomen*. Sternites 3–7 with setae restricted to posterior two-thirds and laterad; sternite 8 strongly arched into preceding segment, lacking setae.

*Terminalia* (Figs 2B, 3B). Gonocoxite subquadrate, posterior inner margin produced into pointed projection, outer margin notched. Gonostylus short, slightly more than half-length of cercus, strongly arched outwards; apex pointed. Parameres distally fused, widest medially, as wide as gonocoxite; extended past posterior margin of epandrium; lateral margins curved ventrally, canal-like medially in ventral view, then tapered rapidly to filamentous, pointed apex projected posterodorsally, extended beyond epandrium; medial structure cradled within canal, protruded ventrally; in lateral view ending at halfway point of longest filament. Gonocoxal apodeme with secondary structure comprising single filament, running along interior of paramere canal, projected ventrally at apex. Cercus trapezoidal; projected posteroventrally; situated within epandrial indentation.

**Female.** Unknown.

#### Immature stages.

Unknown.

#### Additional material examined.

Known only from the type series.

#### Distribution.

Known from the foothills of the southern Andes in Chile (Fig. 24A).

#### Etymology.

*Niphtadownesi* is named in honour of veterinary and medical entomologist J.A. Downes, who collected the first specimen of this species in 1985.

### Niphta halteralis

Taxon classificationAnimaliaDipteraThaumaleidae

(Edwards)

B94E2F84-5170-5AEB-AE9F-01E1821A88BC

Figs 2C , 3C , 4C , 13C, D , 24A , 27F

Austrothaumalea halteralis Edwards, 1930: 114. Stuardo, 1946: 42 (catalogue); Stone, 1966: 1 (catalogue); Arnaud, 1977: 284 (distribution).
Niphta halteris (Edwards): Theischinger 1986: 316 (*lapsis calami*, new combination); McLellan, 1988: 563 (moved to genus Niphta by Theischinger (1986)).

#### Type material examined.

***Holotype*:** ♂, minuten pinned with abdomen mounted in resin, labelled: “Casa Pangue./ 4–10.xii.1926.”; “Austrothaumalea/ halteralis Edw./ F.W. Edwards/ det. 1930.”; “S. Chile:/ Llanquihue prov./ F. & M. Edwards./ B.M. 1927 – 63.”; “HOLO-/ TYPE [white label with red margin]”; “NHMUK010210689”.

#### Recognition.

This species is recognised by its distinct hook-tipped paramere.

#### Redescription.

The redescriptions of *N.halteralis* differ from that of *N.acus* in the following regards:

**Male.***n* = 44.

*Length* 1.3–2.0 mm.

*Colouration* (Fig. 4C). Head dull, dark brown to black; postpronotal lobe, prescutum and mesoscutum shiny, brown, middle of postscutum light brown; scutellum and mediotergite shiny, light brown; pteropleuron light brown with dispersed dark brown markings; base of halter light brown, knob creamy yellow; legs pale brown, tarsi darker; abdomen dark brown, hind margins of tergites whitish; terminalia light brown.

*Head*. Frons with two strong setae. Flagellomeres 1–3 subquadrate, slightly expanded, 0.25 × as wide as next segment, slightly shorter than 2 and 3 combined. Vertex with yellow setae of uniform length, with longer, black orbital setae.

*Thorax*. Antealar ridge bearing single pronounced medial seta flanked by two smaller setae.

*Wing*. Wing length: 2.0–2.9 mm. C and posterior wing margin with fringe of microtrichia; R_2+3_ distinct, situated slightly before middle of R_1_(+R_2+3_); M_1_ and M_2_ straight.

*Abdomen*. Sternites 3–7 rectangular, lacking distinct sclerites, setae restricted to posterior half; sternite 8 strongly arched into preceding segment, lacking setae.

*Terminalia* (Figs 2C, 3C). Gonocoxite subquadrate; posterior inner margin produced into pointed projection; inner margin densely setose; outer margin without notch. Gonostylus short, about as long as cercus, widest at base, strongly tapered along outer margin with rounded apex; apex slightly flanged outward, appearing pointed in lateral view; distal half bearing setae along outer apical margin, a few setae on inner margin. Parameres fused at gonocoxal apodeme, widest medially, as wide as gonocoxite; extended past posterior margin of epandrium; lateral margins curved ventrally forming canal-like structure with median ‘wings’ projected posteriorly, margins feathered; apex with pointed hook-like projection, recurved and projected anteriorly to left. Gonocoxal plate well sclerotised; tongue-like plate extended anteroventrally; gonocoxal apodeme with secondary bridge connected with base of paramere. Cercus trapezoidal; projected posteroventrally.

**Female.***n* = 6.

Similar to male except as follows: *Abdomen*. Tergite 9 noticeably more sclerotised than preceding tergites; sternite 8 well sclerotised.

*Terminalia* (Fig. 13C, D). Hypogynial valve with posterior margin deeply emarginated in ventral view, forming two triangular lobes. Tergite 9 oblong in lateral view, twice as wide as tergite 8. Sternite 9 (genital fork) slender, T-shaped; lateral arms not extended beyond hypogynial valve, Y-shaped; with ventral sclerite in hypogynial valve cleft. Two sclerotised, tube-like structures (perhaps spermathecal pumps) dorsal to base of lateral arms in lateral view; spermathecal receptacles and ducts not observed.

#### Immature stages.

Unknown.

#### Additional material examined.

Chile: Region X (Los Lagos): Camino de Penetracion @ Hwy. 7 sign, 16.xii.2013, 42°07'57.5"S 72°27'45.3"W, seep, sweeping veg., G.R. Curler (1♂, 1♀*); Camino de Penetracion @ km post 125.600, 16.xii.2013, 42°03'33.3"S 72°27'07.4"W, rock seep, G.R. Curler (1♂); Casa Pangue, Llanquihue, 12.1926, R&E Shannon, USNMENT01115811 (1♂, USNM); Ensenada, nr. Baños de Petrohué, 12.i.1985, J.A. Downes (2♂, CNC; 1♂, USNM (USNMENT01115812)); Rte. 215, 12.xii.2016, 40°40'32.4"S 72°17'35.6"W, elev. 252 m, trickle falls, J.K. Moulton & R.J. Pivar (1♂); Rte. U-99, 10.xii.2016, 41°08'09.6"S 72°35'43.3"W, elev. 81 m, roadside falls, J.K. Moulton & R.J. Pivar (15♂; 4♀*); Rte. U-99, 10.xii.2016, 41°08'28.2"S 72°35'16.8"W, elev. 101 m, roadside seeps/creek, J.K. Moulton & R.J. Pivar (11♂, 1♀*); Rte. V-69, 12.xii.2016, 41°26'37.7"S 72°17'42.2"W, elev. 34 m, cascading stream, J.K. Moulton & R.J. Pivar (1♂); Rte. V-69, Puente El Salto, 12.xii.2016, 41°31'29.2"S 72°17'14.6"W, elev. 37 m, splash zone above falls, J.K. Moulton & R.J. Pivar (2♂); Region XIV (Los Ríos): Antilhue, Rte. T-35, 9.xii.2016, 39°49'09.8"S 72°56'22.6"W, elev. 40 m, roadside creek, J.K. Moulton & R.J. Pivar (3♂); Valdivia, Los Ulmos Rd., 15.i.1985, J.A. Downes (5♂, CNC).

#### Distribution.

Known from both the Andes and Chilean Coastal Range in southern Chile (Fig. 24A).

#### Bionomics.

This species appears restricted predominantly to low elevations in the Valdivian temperate rain forest.

### Niphta mapuche

Taxon classificationAnimaliaDipteraThaumaleidae

Pivar sp. nov.

F6C00F4B-0894-51EB-9C4F-8FC2B3C87AA7

http://zoobank.org/DD325D3C-7AAF-4FB5-9060-42CBCD462511

Figs 2D , 3D , 4D , 13E, F , 20C, D , 24A

#### Type material.

***Holotype*:** ♂, glued to point with abdomen in glycerine microvial pinned beneath, labelled: “Chile: Region IX (Araucanía)/ Rte. S-365, 14.xii.2016/ 38°46'27.0"S 71°36'51.0"W/ elev. 809 m, creek/small falls/ J.K. Moulton & R.J. Pivar”; “HOLOTYPE/ *Niphta*/ *mapuche*/ Pivar [red label]” (CNC). ***Allotype***: ♀, same label data as holotype (CNC). ***Paratypes***: Chile: Region VIII (Bío Bío): Rte. Q-61, 8.xii.2016, 37°48'22.8"S 71°40'46.6"W, elev. 379 m, cascading creek, J.K. Moulton & R.J. Pivar (2♂, 1♀*); Rte. Q-689, 8.xii.2016, 37°54'55.6"S 71°35'43.2"W, elev. 552 m, cascading creek, J.K. Moulton & R.J. Pivar (2♀*); Region IX (Araucanía): nr. Tolhuaca N.P., Rte. 71, 15.xii.2016, 38°13'23.5"S 71°49'07.8"W, elev. 934 m, stream, J.K. Moulton & R.J. Pivar (1♂); Rte. S-365, 14.xii.2016, 38°46'27.0"S 71°36'51.0"W, elev. 809 m, creek/small falls, J.K. Moulton & R.J. Pivar (4♂, 1♀*); Salto El Léon (spray zone at base), 2.xii.2013, 39°25'10.9"S 71°45'42.3"W, elev. 760 m, madicolous habitats, G.W. Courtney (CH13080) (3♀*, 4 pupal exuviae); same label data as previous except, 3.x.2007 (8 larvae); upper Rio Malleco @ Rd Xing, 25.xii.2013, 38°13'20.0"S 71°44'40.8"W, elev. 1040 m, from riffle rocks, G.W. Courtney (CH13–030) (7 larvae*); Region X (Los Lagos): Parque Nacional Alerce Andino, culvert falls above Sargazo GS, 1.xii.2008, 41°30'31.8"S 72°37'13.8"W, elev. 335 m, G.W. Courtney (CH08–23) (4 larvae*); Region XIV (Los Ríos): Estero Altura Pazas on Cosh-Liq Rd., 1.xii.2013, 39°44'43.8"S 71°56'22.14"W, elev. 385 m, on wetted rock, G.W. Courtney (CH13–079) (6 larvae*); Rte. T-29, Puente Altura Pazas, 14.xii.2016, 39°44'43.6"S 71°56'24.4"W, elev. 363 m, cascading creek, J.K. Moulton & R.J. Pivar (2♂, 2♀*).

#### Recognition.

This species is recognised by the paramere being mostly hidden within the epandrium in lateral view, giving the paramere a two-filament appearance. The gonostylus is less recurved than that of *N.downesi*.

#### Description.

The descriptions of *N.mapuche* differ from that of *N.acus* in the following regards:

**Male.***n* = 10.

*Length* 1.4–2.5 mm.

*Colouration* (Fig. 4D). Head dull, black; pronotum and postpronotum brown; prescutum and mesoscutum shiny, blackish brown, pleura brown; katepisternum brown with blackish brown margins; base of halter light brown turning black medially, knob creamy yellow; legs ranging from pale brown to blackish brown; abdomen blackish brown, posterior margins creamy; terminalia variable in colour from blackish brown to grey.

*Head*. Flagellomeres 1–3 subquadrate, 1 expanded, 2 × as wide as next segment, as long as 2 and 3 combined.

*Abdomen*. Abdominal sternite 2 with few setae restricted to laterad on posterior third; sternites 3–7 with setae restricted to lateral margins and middle third; sternite 8 with three or fewer setae medially and on lateral margins.

*Terminalia* (Figs 2D, 3D). Gonocoxite subquadrate; posterior inner margin slightly produced into pointed projection, outer margin notched. Gonostylus short, less than half-length of cercus, strongly curved lateroventrally throughout; apex pointed; outer margin bearing laterally directed setae. Parameres distally fused, widest at point of fusion; not reaching posterior margin of epandrium; dividing into two pointed, filamentous projections medially; shorter ventral filament not extended beyond gonostyli, apex directed posteriorly; longer dorsal filament not extended to posterior margin of epandrium, at most slightly beyond base of cercus, apex projected slightly posterodorsally. Gonocoxal plate broad, well sclerotised, tongue-like plate extending anteroventrally; gonocoxal apodeme with secondary structure comprising single short, indistinct filament, running along interior of paramere, ending before apical margin of gonostylus. Cercus trapezoidal.

**Female.***n* = 10.

Similar to male except as follows: *Terminalia* (Fig. 13E, F). Hypogynial valve not projected beyond tergite 9; posterior margin deeply emarginated in ventral view, forming two triangular lobes; lateral margin sclerotised at base. Tergite 9 triangular in lateral view. Sternite 9 (genital fork) slender, Y-shaped; lateral arms not extended beyond hypogynial valve, fused distally forming rounded posterior margin; lightly sclerotised ventral plate at base of hypogynial valve. Two sclerotised, tube-like structures dorsal to genital fork, spermathecal pumps perhaps; spermathecal receptacles and ducts not observed.

**Pupa.***n* = 4 (not figured due to condition of specimens).

*Length* 3.5–4.0 mm.

*Head*. Setae not observed.

*Thorax*. Hindleg concealed behind wing sheath, only apex visible between apex of foreleg and wing sheath, slightly shorter than foreleg, but longer than wing sheath. Wing sheaths with large tubercle at base, setae not visible.

*Abdomen*. Setae not visible on tergites 1–8.

**Larva.***n* = 17.

*Length of final instar* 6.3–6.7 mm.

*Colouration*. Head capsule variable, ranging from light brown to black. Body mottled brown and grey, possibly pale brown to creamy; cream coloured ventrally.

*Head capsule* (Fig. 20C, D). Tubercles below and beside eye bifurcate; two tubercles between ecdysial line, upper tubercle bi- or trifurcate.

*Abdomen*. Tergites 1–7 with lateral adhesive structure swelling bearing five setae, two lateral, three basal.

#### Additional material examined.

Known only from the type series.

#### Distribution.

Known from the southern Andes of Chile (Fig. 24A).

#### Etymology.

This species is named after the Mapuche (*mapu*, land, *che*, people) indigenous peoples, who since ~ 500 B.C., have inhabited the regions of southern Chile, where *N.mapuche* is known.

#### Bionomics.

This is a mid-elevation species. Adults were observed flying around and resting on leaf tips, roughly two meters from the nearest splash zones. Larvae and pupae have ventral adhesive structures and were collected only from rocky substrates at the margin of a waterfall.

##### The *N.nudipennis* group

### Niphta bifurcata

Taxon classificationAnimaliaDipteraThaumaleidae

Pivar and Moulton sp. nov.

9EF2ABFC-ED8F-5131-97FB-BAD6DCC06080

http://zoobank.org/2C174B79-C941-4878-9A41-AC43414033FC

**Figure 9.**
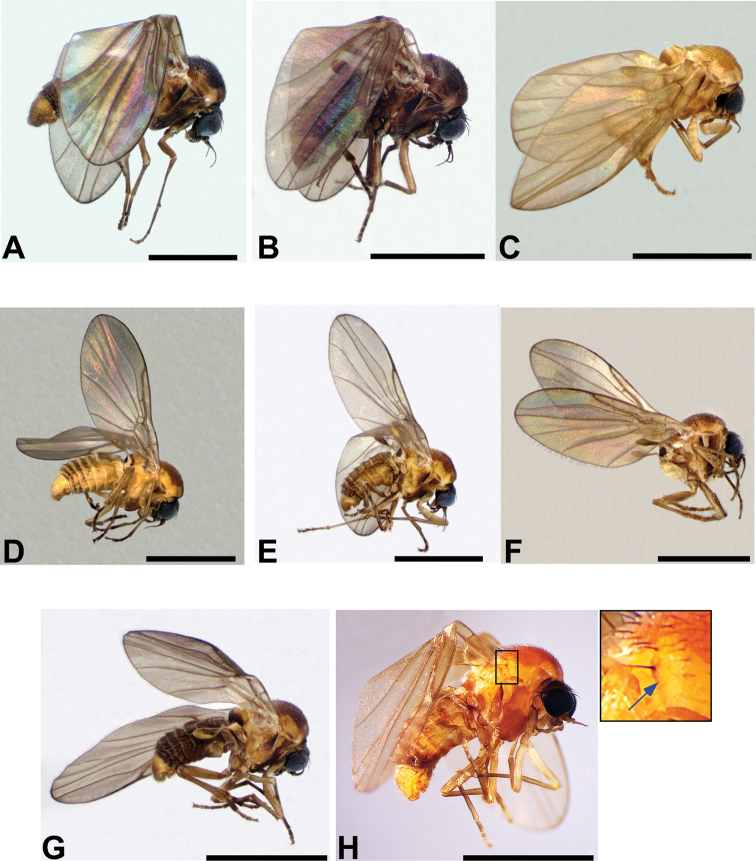
Adult lateral habitus micrographs of the *Niphtanudipennis* group **A***N.bifurcata* sp. nov. (♂) **B***N.brunnea* sp. nov. (♂) **C***N.courtneyi* sp. nov. (♀), abdomen dissected **D***N.daniellae* sp. nov. (♂) **E***N.eurydactyla* sp. nov. (♂) **F***N.bispinosa* sp. nov. (♂), abdomen dissected **G***N.nudipennis* (♂) **H***N.courtneyi* sp. nov. (♂), inset with arrow indicating antealar ridge. Scale bars: 1.0 mm.

**Figure 10.**
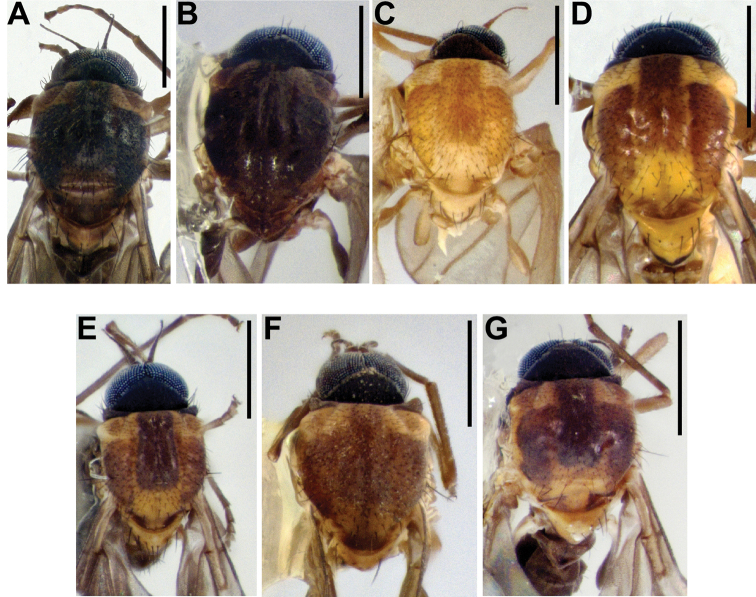
Adult dorsal habitus micrographs of the *Niphtanudipennis* group **A***N.bifurcata* sp. nov. (♂) **B***N.brunnea* sp. nov. (♂) **C***N.courtneyi* sp. nov. (♀), abdomen dissected **D***N.daniellae* sp. nov. (♂) **E***N.eurydactyla* sp. nov. (♂) **F***N.bispinosa* sp. nov. (♂), abdomen dissected **G***N.nudipennis* (♂) Scale bars: 1.0 mm.

**Figure 11.**
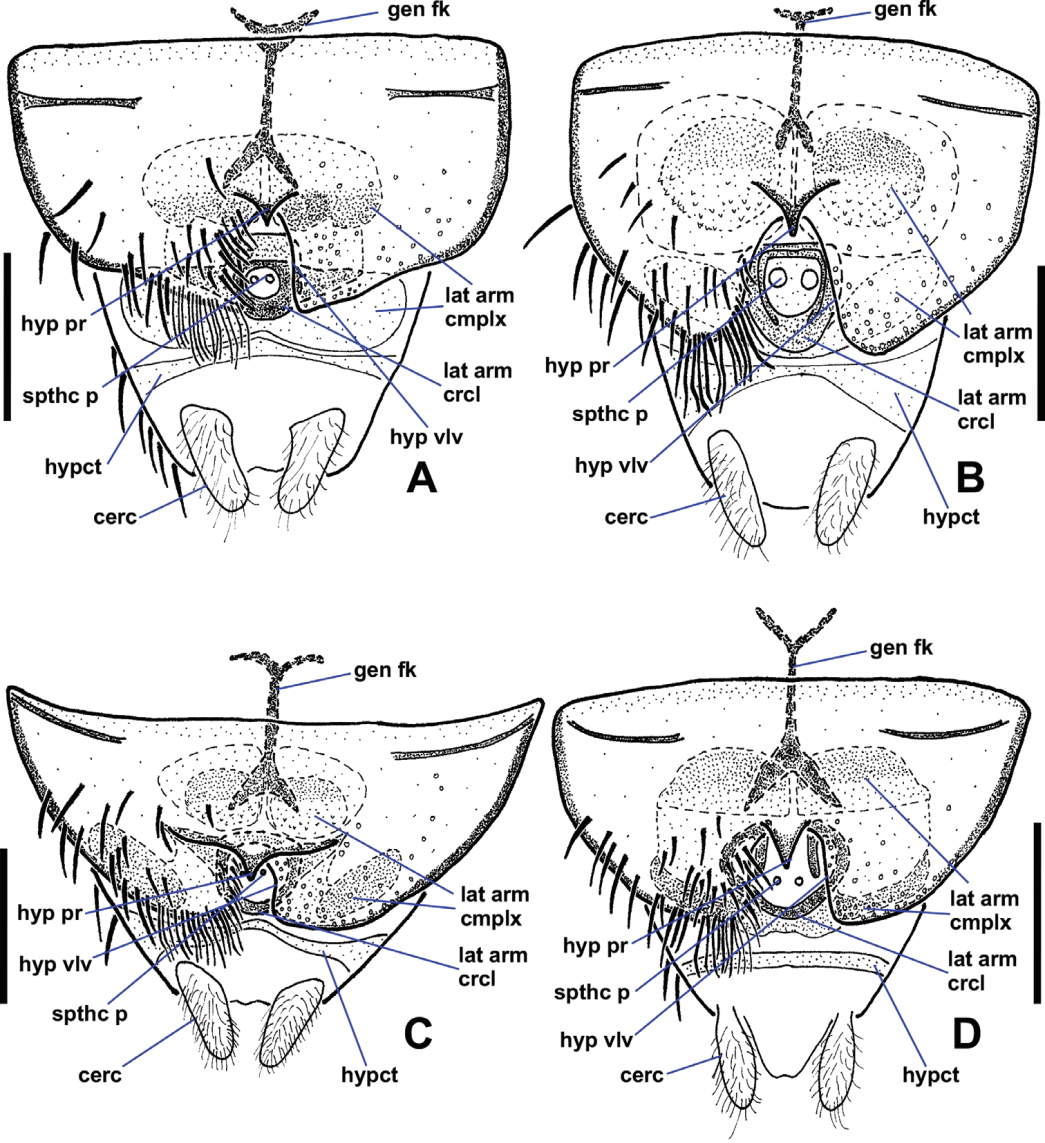
Ventral views of female *Niphtanudipennis* group terminalia **A***N.bifurcata* sp. nov. **B***N.courtneyi* sp. nov. **C***N.bispinosa* sp. nov. **D***N.nudipennis*. Abbreviations: cerc, cercus; gen fk, genital fork; hyp pr, hypogynial protuberance; hyp vlv, hypogynial valve; hypct, hypoproct; lat arm crcl, lateral arm circle; lat arm cmplx, lateral arm complex; spthc p, spermathecal pump. Scale bars: 0.1 mm.

**Figure 12.**
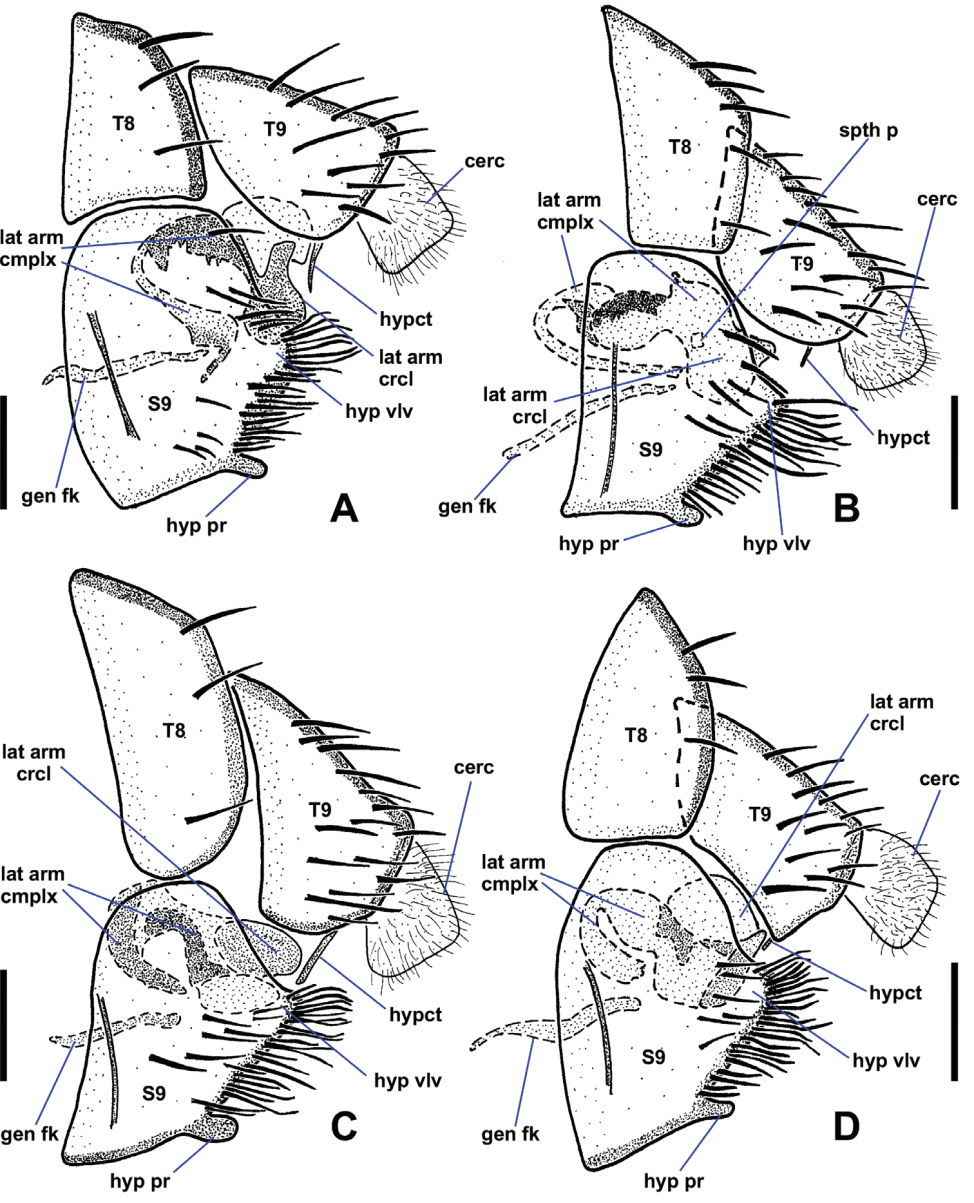
Lateral views of female *Niphtanudipennis* group terminalia **A***N.bifurcata* sp. nov. **B***N.courtneyi* sp. nov. **C***N.bispinosa* sp. nov. **D***N.nudipennis*. Abbreviations: cerc, cercus; gen fk, genital fork; hyp pr, hypogynial protuberance; hyp vlv, hypogynial valve; hypct, hypoproct; lat arm crcl, lateral arm circle; lat arm cmplx, lateral arm complex; S, sternite; spthc p, spermathecal pump; T, tergite. Scale bars: 0.1 mm.

Figs 6A , 8A , 9A , 10A , 11A , 12A , 24B

#### Type material.

***Holotype*:** ♂, glued to point with abdomen in glycerine microvial pinned beneath, labelled: “Chile: Region XIV (Los Ríos)/ Antilhue, Rte. T-35, 9.xii.2016/ 39°49'09.8"S 72°56'22.6"W/ elev. 40 m, roadside creek,/ J.K. Moulton & R.J. Pivar”; “HOLOTYPE/ *Niphta*/ *bifurcata*/ Pivar & Moulton [red label]” (CNC). ***Allotype***: ♀*, same data as holotype (CNC). ***Paratypes***: Chile: Region XIV (Los Ríos): same data as holotype (2♂).

#### Recognition.

This species is recognised by the bifurcate, posterior apex of the cheliform gonostylus and the bifurcate anterior projection of the gonocoxite. It is darker in colouration compared to the closely related *N.courtneyi*.

#### Description.

**Male.***n* = 3.

*Length* 1.7–1.9 mm.

*Colouration* (Figs 9A, 10A). Head dull, dark brown; pronotum and postpronotum dark brown; postpronotal lobe and lateral margins of prescutum light brown; scutum shiny with three distinct dark brown stripes, pleura light brown; postscutum brown; scutellum shiny, light brown; mediotergite shiny, anterior half light brown, posterior half brown; katepisternum mainly dark brown, light brown near coxa 1; paratergite brown; remaining pteropleuron mainly brown with dispersed light brown and black markings; base of halter pale brown, knob pale yellow; legs pale brown, apex of tarsi darker; abdomen brown; terminalia pale brown.

*Head*. Eyes above antennae broadly joined, with small triangular frons visible above antennae; frons with two strong setae. Flagellomeres 1–3 subquadrate, 1 expanded, twice as wide as next segment, shorter in length than 2 and 3 combined; flagellomeres 4–10 cylindrical, becoming progressively thinner and elongate. Vertex with black setae of uniform length, with longer, black orbital setae.

*Thorax*. Mesoscutum with prominent antealar ridge, bearing three setae, middle seta most pronounced. Scutum clothed dorsally in short, black setulae; notopleural, supra-alar and postsutural setae long, black. Pteropleuron bare. All legs with tarsi simple.

*Wing*. Wing length: 2.1–2.4 mm. Infuscate throughout, apex somewhat narrowed; C fringed in small setulae, with widely spaced microtrichia; posterior wing margin with closely spaced fringe of microtrichia; Sc incomplete; R_1_ and R_1_(+R_2+3_) with three weakenings or depigmented gaps, first slightly beyond R_2+3_, second and third closely approximated, near C; microtrichia of R_1_(+R_2+3_) confined to base near humeral crossvein, remaining veins bare; R flexed into cell br; R_2+3_ distinct, situated in basal third of R_1_(+R_2+3_); bend in R_4+5_ strong; R_4+5_ and M_1_ running parallel toward margin; M_1_ straight; M_2_ with gentle bend in apical third; M_4_ with slight bend.

*Abdomen*. Abdominal sternite 1 narrow, spectacle-shaped; sternite 2 reduced to slender median sclerite, a few setae restricted to posterior third; sternites 3–7 rectangular, lightly sclerotised, setae restricted to posterior half; sternite 8 strongly reduced, anterior margin well sclerotised, arched slightly into preceding segment, a few setae restricted to laterad.

*Terminalia* (Figs 6A, 8A). Epandrium quadrate in ventral view, posterior margin rounded, with medial cleft; long, extended beyond gonostyli; without lobes or projections. Gonocoxites oblong, longer than wide; anterior margin rounded, somewhat expanded dorsally behind gonocoxal plate, not closely approximated; with two spine-like projections; anterior projection wide, bifurcate; posterior projection long, slender, slightly sinuous, tapered to single apex, nearly twice as long as anterior projection; inner margin with numerous long, thin setae. Gonostylus cheliform, dorsoventrally flattened anteriorly, swollen posteriorly; anterior apex with a few setae; posterior apex bifurcate, setose. Parameres medially fused, attached basally to arms of gonocoxal plate; divided distally into dorsal parameral apodeme and ventral arm; ventral arm projected anteroventrally toward gonocoxal plate, strongly curved anteriorly, sickle-shaped, surface textured with tiny bumps, except for smooth apex; ventral arm extends posteroventrally presumably to aid in copulation; when retracted, rests ventrally between dorsal arm of gonocoxal plate and dorsal to anterior gonocoxal projection. Gonocoxal plate broad, well sclerotised; anterior margin subquadrate, basal margin cleft; pair of dorsal arms connected to parameres; medial aedeagal guide projected ventrally between posterior margins of gonocoxites, well sclerotised, comprising two parts: anterior Y-shaped structure with five finger-like projections protruded from posterior margin and dorsal triangular plate. Cercus ovoid, only slightly visible in lateral view; projected anteroventrally; situated within epandrial indentation.

**Female.***n* = 1.

Similar to male except as follows: *Abdomen*. Tergite 9 noticeably more sclerotised than preceding tergites; sternite 8 well sclerotised, with distinct blunt projection at base of hypogynial valve. *Terminalia* (Figs 11A, 12A). Hypogynial valve not projected beyond tergite 9; posterior margin deeply cleft in ventral view, forming two triangular lobes; lobes densely setose, with both stout, thickened setae and thinner, long setae with slight apical bend; hypogynial protuberance between valves. Tergite 9 subquadrate in lateral view, 1.5 × as wide as tergite 8, lacking lateral projections. Sternite 9 (genital fork) slender, Y-shaped at both ends; lateral arms forming complex of highly modified structures: medial heavily sclerotised circular opening, dorsal to posterior cleft of hypogynial valve, with pair of lateral sclerotised triangular expansions; triangular expansions expanded dorsally into pair of circular plates, those further expanded anteriorly into pair of heavily sclerotised plates, strongly recurved posteroventrally toward circular opening, remaining dorsal to genital fork; dorsal surface of recurved plates with tiny grooves and indentations. Hypoproct lightly sclerotised, narrow. Cercus quadrate, projected posteroventrally; bearing numerous setae. Spermathecae not observed; two spermathecal ducts visible in centre of lateral arm circle.

#### Immature stages.

Unknown.

#### Additional material examined.

Known only from the type series.

#### Distribution.

Known only from the type locality in the Chilean Coastal Range (Fig. 24B).

#### Etymology.

*Niphtabifurcata* is named in reference to the posterior apex of the gonostylus and the anterior projection of the gonocoxite, both of which are bifurcate.

### Niphta bispinosa

Taxon classificationAnimaliaDipteraThaumaleidae

Pivar and Sinclair sp. nov.

F969A23F-E28D-5676-B17F-9C9C60CF5032

http://zoobank.org/9A9B5605-3AD0-4019-8019-BF8C47828DA5

Figs 6C , 8C , 9F , 10F , 11C , 12C , 24B , 27B, D

#### Type material.

***Holotype*:** ♂, glued to point with abdomen in glycerine microvial pinned beneath, labelled: “Chile: Region VII (Maule)/ Los Queñes, Rte. J-25,/ 6.xii.2016, 34°59'46.7"S 70°49'19.2"W/ elev. 679 m, cascading creek,/ J.K. Moulton & R.J. Pivar”; “HOLOTYPE/ *Niphta*/ *bispinosa*/ Pivar & Sinclair [red label]” (CNC). ***Allotype***: ♀*, same data as holotype (CNC). ***Paratype***: Chile: Region VII (Maule): Los Queñes, Rte. J-25, 6.xii.2016, 34°59'48.8"S 70°48'37.0"W, elev. 684 m, seep, J.K. Moulton & R.J. Pivar (1♀*).

#### Recognition.

This species is recognised by the cheliform gonostylus with non-bifurcate apices and the gonocoxite with two projections, the anterior one long and bifurcate, the posterior one small, tooth-like. It is lighter in colouration than the closely related *N.brunnea*.

**Figure 13.**
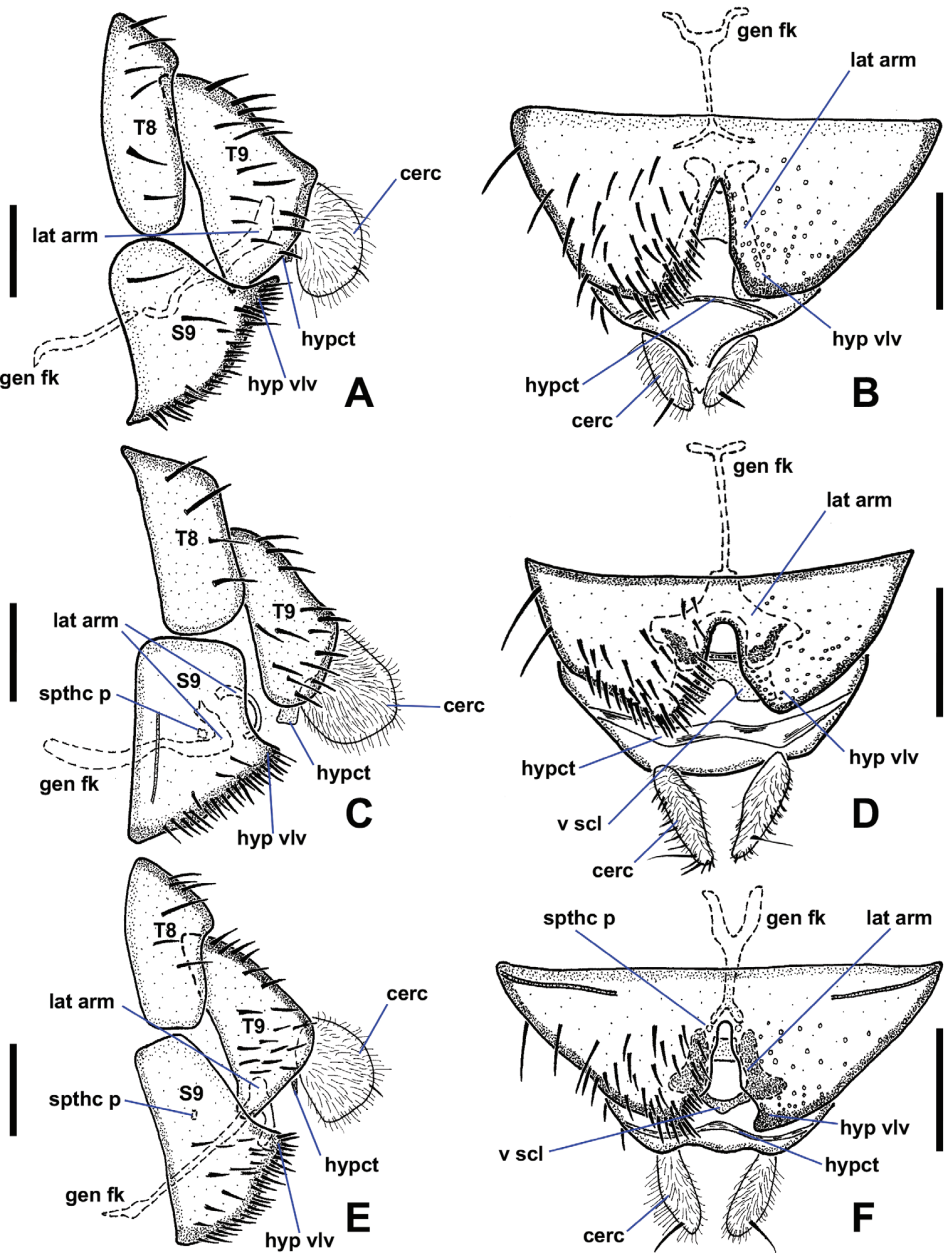
Female terminalia of the *Niphtahalteralis* group **A** lateral, *N.acus* sp. nov. **B** ventral, *N.acus* sp. nov. **C** lateral, *N.halteralis***D** ventral, *N.halteralis***E** lateral, *N.mapuche* sp. nov. **F** ventral, *N.mapuche* sp. nov. Abbreviations: cerc, cercus; gen fk, genital fork; hyp vlv, hypogynial valve; hypct, hypoproct; lat arm, lateral arm; S, sternite; spthc p, spermathecal pump; T, tergite; v scl, ventral sclerite. Scale bars: 0.1 mm.

#### Description.

The descriptions of *N.bispinosa* differ from that of *N.bifurcata* in the following regards:

**Male.***n* = 1.

*Length* 1.9–2.4 mm.

*Colouration* (Figs 9F, 10F). Pronotum and postpronotum brown; postpronotal lobe and lateral margins of prescutum pale yellow; remaining scutum shiny, brown, pleura yellow; postscutum with medial brown spot above scutoscutellar suture, encircled by yellowish brown margin; scutellum shiny, yellow; mediotergite shiny, dark brown except anterior margin yellow; katepisternum mainly brown, lighter near anterior spiracle; remaining pteropleuron mainly pale yellow with dispersed brown markings; base of halter yellow, distal half of stalk and knob grey; legs pale yellow, apex of tarsi darker; terminalia light brown.

*Head*. Frons with three strong setae. Flagellomere 1 expanded, 1.5 × as wide as next segment, equal in length to 2 and 3 combined.

*Wing*. Wing length: 2.0–2.4 mm. Lightly infuscate throughout; bend in R_4+5_ gentle.

*Terminalia* (Figs 6C, 8C). Posterior margin of epandrium rounded, with narrow medial cleft. Gonocoxites oblong, wider than long; anterior margin rounded, somewhat expanded dorsally behind gonocoxal plate, not closely approximated; with two spine-like projections; anterior projection bifurcate, projected posteriorly, 3 × longer than posterior projection; posterior projection short, tooth-like; margin around gonostylus setose. Gonostylus cheliform, dorsoventrally flattened, margins concave creating bowl-like appearance; anterior apex with a few indistinct setae, posterior apex with strong setae. Parameres medially fused, attached basally to arms of gonocoxal plate; divided distally into dorsal parameral apodeme and ventral arm; ventral arm projected anteroventrally toward gonocoxal plate, strongly curved anteriorly, blade-like, ventral margin serrate; ventral arm extended posteroventrally presumably to aid in copulation; when retracted, rests ventrally between dorsal arm of gonocoxal plate and inner margin of gonocoxite, apex reaching base of anterior gonocoxal projection. Gonocoxal plate broad, well sclerotised; anterior margin subquadrate, basal margin cleft; pair of dorsal arms connected to parameres; medial aedeagal guide projected ventrally between gonostyli, well sclerotised, comprising two parts: anterior Y-shaped structure and posterior triangular plate. Cercus prominent, ovoid.

**Female.***n* = 2.

Similar to male except as follows: *Terminalia* (Figs 11C, 12C). Posterior margin of hypogynial valve deeply cleft in ventral view, forming two quadrate lobes. Tergite 9 twice as wide as tergite 8. Sternite 9 (genital fork) slender, Y-shaped at both ends; lateral arms forming complex of highly modified structures: medial heavily sclerotised circular opening, dorsal to posterior opening of hypogynial valve, with pair of lateral sclerotised triangular expansions. Spermathecae not observed.

#### Immature stages.

Unknown.

#### Additional material examined.

Known only from the type series.

#### Distribution.

Known only from the type locality in central Chile (Fig. 24B).

#### Etymology.

*Niphtabispinosa* is named in reference to the two projections from the gonocoxite.

### Niphta brunnea

Taxon classificationAnimaliaDipteraThaumaleidae

Pivar sp. nov.

D9103CED-4302-5A22-A61F-64491363B418

http://zoobank.org/ED8B78EE-F799-4B34-B465-E47C578BC679

Figs 6D, 8D, 9B, 10B, 14A, 15A, 16A, 17A, 18A, 19A, 21A, B, 22, 23, 24B, 26A–C

#### Type material.

***Holotype*:** ♂, glued to point with abdomen in glycerine microvial pinned beneath, labelled: “Chile: Region IX (Araucanía)/ Rte. 71, 15.xii.2016/ 38°14'20.6"S 71°53'46.6"W,/ elev. 953 m, roadside seeps,/ J.K. Moulton & R.J. Pivar”; “HOLOTYPE/ *Niphta*/ *brunnea*/ Pivar [red label]” (CNC). ***Paratypes***: Chile: Region VIII (Bío Bío): Rte. Q-61, 8.xii.2016, 37°49'14.2"S 71°40'34.1"W, elev. 366 m, creek, J.K. Moulton & R.J. Pivar (1♂); Rte. Q-61, Estero Agua Blanca 8.xii.2016, 37°46'30.8"S 71°42'03.9"W, elev. 552 m, vegetation near splash zones, J.K. Moulton & R.J. Pivar (1♂); Region IX (Araucanía): same data as holotype (3♂); same data as holotype except, larvae/pupae on foliage in splash zone (6 larvae*, 5 pupae, 3 pupal exuviae).

#### Recognition.

This species is recognised by the cheliform gonostylus bearing non-bifurcate apices and the gonocoxite with three projections: two that are nearly equal in size and the third, much smaller and inconspicuous, situated at the base of the anterior one. It is darker in colouration compared to the closely related *N.bispinosa*, and the darkest of the *N.nudipennis* group.

#### Description.

The descriptions of *N.brunnea* differ from that of *N.bifurcata* in the following regards:

**Male.***n* = 5.

*Length* 1.6–1.9 mm.

*Colouration* (Figs 9B, 10B). Pronotum and postpronotum brown; remaining scutum shiny, brown, pleura light brown; scutellum shiny, brown; mediotergite shiny, anterior half light brown, posterior half dark brown; katepisternum mainly brown, lighter near anterior spiracle; remaining pteropleuron mainly brown with dispersed markings of dark/light brown; base of halter creamy, distal half of stalk and knob light brown; abdomen brown; terminalia light brown.

*Head*. Flagellomere 1 expanded, 1.5 × as wide as next segment, shorter in length than 2 and 3 combined.

*Wing*. Wing length: 1.9–2.2 mm.

*Terminalia* (Figs 6D, 8D). Epandrium quadrate in ventral view, posterior margin rounded, with narrow medial cleft; long, extended beyond gonostyli; without lobes or projections. Gonocoxites oblong, longer than wide; anterior margin rounded, somewhat expanded dorsally, not closely approximated; with three spine-like projections; large anterior projection nearly equal in length to posterior projection, gradually tapered toward apex; smaller anterior projection positioned somewhat anterior to large tooth, may be small and inconspicuous in some specimens; posterior projection strongly tapered toward apex, some specimens with second basal tooth on outer margin of projection; margin around anterior projection with long, thin setae. Gonostylus cheliform, dorsoventrally flattened, margins concave creating bowl-like appearance; anterior apex with a few indistinct setae, posterior margin with strong setae. Parameres medially fused, attached basally to arms of gonocoxal plate; divided distally into dorsal parameral apodeme and ventral arm; ventral arm projected anteroventrally toward gonocoxal plate, knife-shaped, ventral margin serrate; when retracted, resting ventrally between dorsal arm of gonocoxal plate and inner margin of gonocoxite, apex reaching base of anterior gonocoxal projection. Gonocoxal plate broad, well sclerotised; anterior margin triangular, basal margin cleft; pair of dorsal arms connect to parameres; medial aedeagal guide projected ventrally between gonostyli, well sclerotised, consisting of two parts, anterior structure with five projections and posterior rounded plate bearing minute setulae. Cercus ovoid, only slightly visible in lateral view; projected anteroventrally; situated within epandrial indentation.

**Figure 14.**
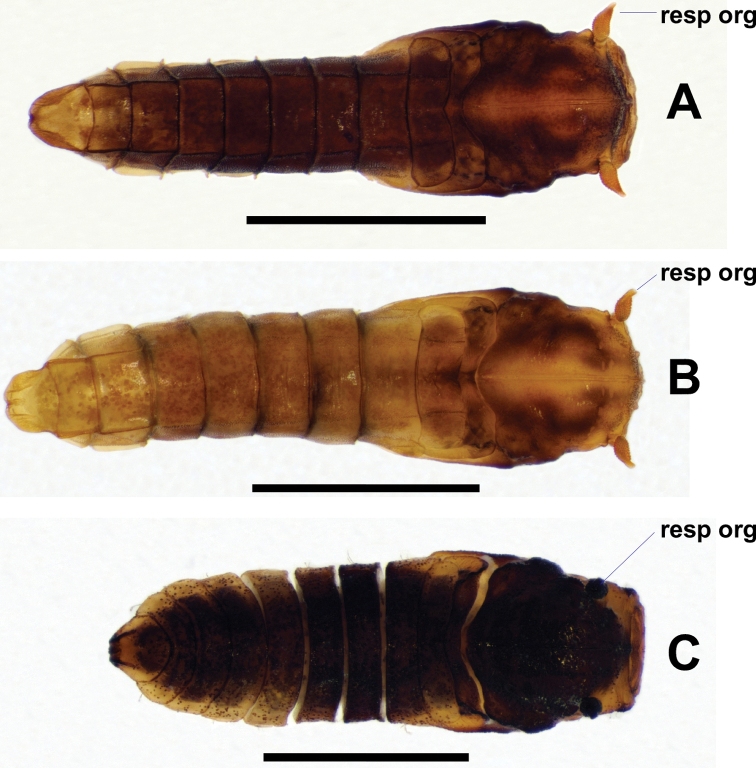
Dorsal views of *Niphta* pupae **A***N.brunnea* sp. nov. **B***N.nudipennis***C***N.acus* sp. nov. Abbreviations: resp org, respiratory organ. Scale bars: 1.0 mm.

**Figure 15.**
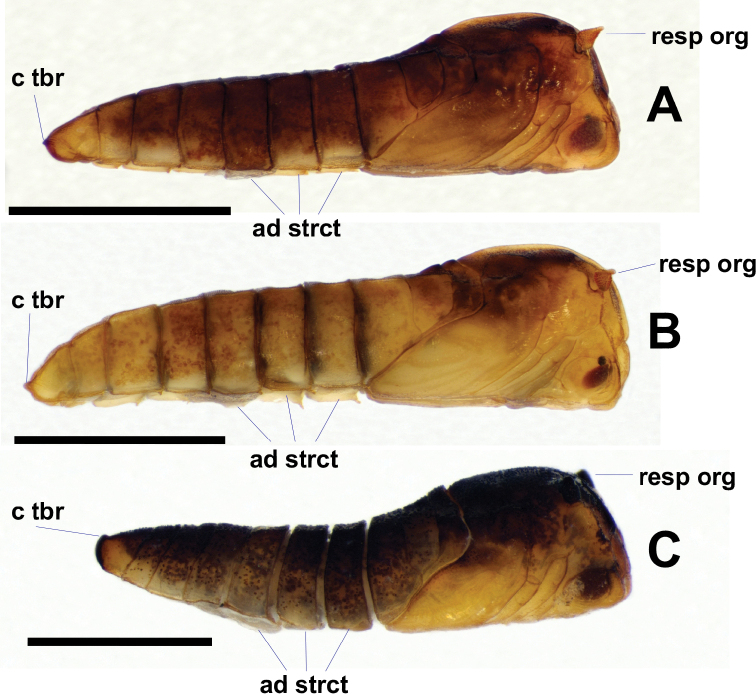
Lateral views of *Niphta* pupae **A***N.brunnea* sp. nov. **B***N.nudipennis***C***N.acus* sp. nov. Abbreviations: ad strct, adhesive structure; c tbr, caudal tubercle; resp org, respiratory organ. Scale bars: 1.0 mm.

**Female.** Unknown.

**Figure 16.**
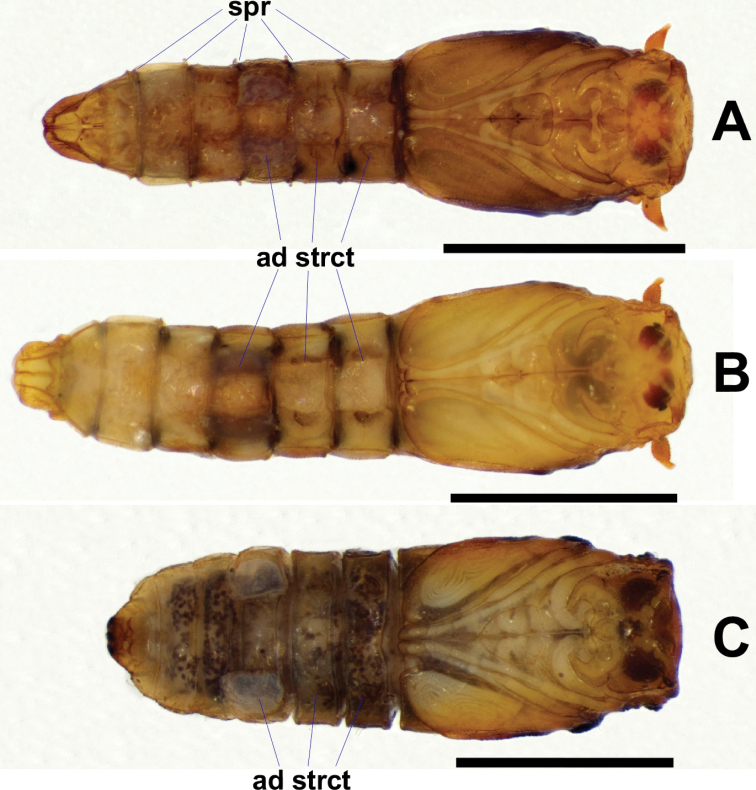
Ventral views of *Niphta* pupae **A***N.brunnea* sp. nov. **B***N.nudipennis***C***N.acus* sp. nov. Abbreviations: ad strct, adhesive structure; spr, spiracle. Scale bars: 1.0 mm.

**Pupa.***n* = 6 (Figs 14A, 15A, 16A, 22).

*Length* 2.7–2.9 mm.

*Colouration*. Brown; sometimes with black spot above eyes in developing adult.

*Head*. Maxillary sheath short, posteromedially directed, gently tapered toward truncate apex, apices of palpi separated medially; devoid of tubercles and setae.

*Thorax*. 1.5 × wider than abdomen at widest point. Foreleg sheath projected straight and slightly beyond wing sheaths, reaching hind margin of sternite 2; anterior half of midleg visible anterior to wing sheath, then hidden behind foreleg, not projected beyond wing sheath; hindleg concealed beneath wing sheath, only small triangular apex visible between apex of foreleg and wing sheath, not extended beyond wing sheath. Wing sheath extended to posterior margin of abdominal sternite 2. Respiratory organ slightly shorter than maxillary sheath, broadest subapically; ovate, slightly arched medially, tapered toward apex; spiracular openings encircling apex; stalk thin, emerging from small tubercle. Tubercle situated posterodorsally to respiratory organ, rounded, projected laterally; apex nearly touching or touching respiratory organ. Thorax devoid of setae.

*Abdomen*. Subcylindrical, evenly tapered toward caudal segment. Spiracles well developed, distinct on segments 3–7, projected anterodorsolaterally; all bearing minute spine-like setulae. Segment 8 with short lateral projection, less than half length of preceding spiracles, projected dorsolaterally. Tergites 1–8 quadrate, devoid of setae, with pair of dorsolateral ridges and faint medial transverse groove, groove not meeting dorsolateral ridges. Tergite 9 rounded, posterior margin emarginated, laterally compressed compared to preceding segments; small, rounded projection pointing posteriorly in lateral view. Sternites 3–8 rectangular, with row of faint setulae along anterior margin, not connecting to lateral margin; lateral margins crenulate, lacking setae. Sternites 3 and 4 bearing pair of small lateral adhesive structures, sternite 5 bearing pair of large lateral adhesive structures on lateral margin. Caudal sternite triangular, with medial sclerotised groove and pair of medial rounded, posteromedially projected lobes; posterior margin with medial longitudinal ridge; without distinct caudal hooks.

**Figure 17.**
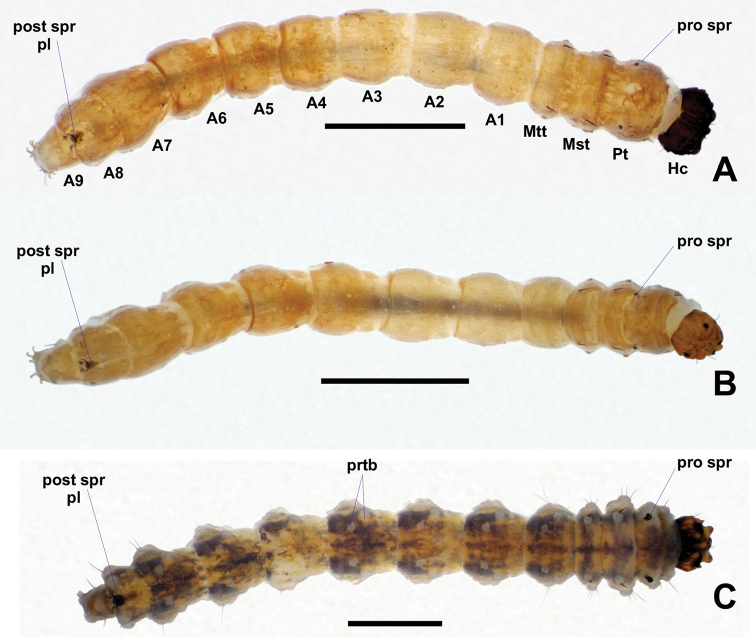
Dorsal views of *Niphta* larvae **A***N.brunnea* sp. nov. **B***N.nudipennis***C***N.acus* sp. nov. Abbreviations: A, abdominal segment; Hc, head capsule; Mst, mesothorax; Mtt, metathorax; Pt, prothorax; pro spr, prothoracic spiracle; prtb, protuberance; post spr pl, posterior spiracular plate. Scale bars: 1.0 mm.

**Figure 18.**
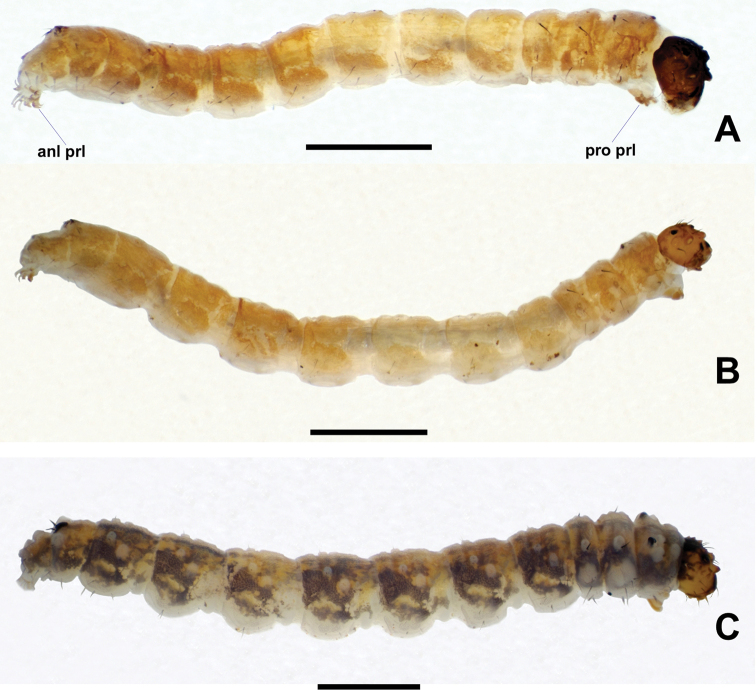
Lateral views of *Niphta* larvae **A***N.brunnea* sp. nov. **B***N.nudipennis***C***N.acus* sp. nov. Abbreviations: anl prl, anal proleg; pro prl, prothoracic proleg. Scale bars: 1.0 mm.

**Figure 19.**
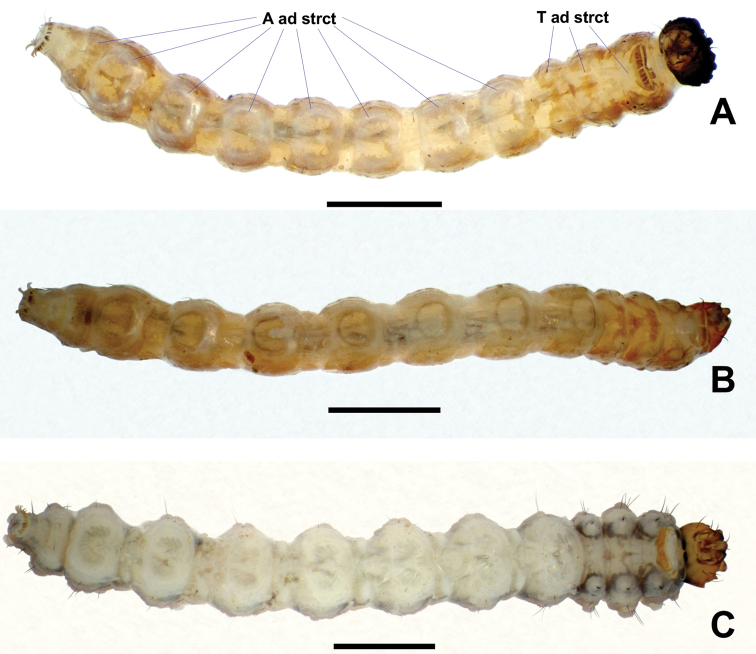
Ventral views of *Niphta* larvae **A***N.brunnea* sp. nov. **B***N.nudipennis***C***N.acus* sp. nov. Abbreviations: A ad strct, abdominal adhesive structure; T ad strct, thoracic adhesive structure. Scale bars: 1.0 mm.

**Larva.***n* = 6 (Figs 17A, 18A, 19A, 21A, B, 23).

*Length of final instar* 4.8–5.1 mm.

*Colouration*. Head capsule pale brown, anterolateral margin of ecdysial line black. Body creamy brown.

*Head capsule* (Fig. 28A, B). Two large, circular eye spots, elevated on tubercle; antenna with three finger-like processes, elevated on largest tubercle; with five pairs of smaller tubercles outside of ecdysial lines (not including antennal and ocular tubercle); single tubercle between ecdysial lines, about same size as ocular tubercle; 15 pairs of unbranched setae; six sensory pits (13, 14, 18, 19, 20, 21), sensory pit 13 above antennal tubercle.

*Thorax*. Prothorax with single pair of protuberances bearing single spiracle; spiracular protuberance bearing one pair of dorsal setae anterior to spiracle and single dorsolateral seta; three mid-lateral setae, two long, one short and fine; two closely approximated setae near base of prothoracic leg (Keilin’s organ). Mesothorax and metathorax with pair of small dorsolateral protuberances and pair of large lateral protuberances; mesothoracic dorsolateral protuberance bearing single thickened seta, metathoracic lateral protuberance bearing pair of closely approximated setae, anterior seta thickened and longer than posterior seta; lateral protuberance on both segments bearing three setae, two short, one long; single long seta slightly ventral to lateral protuberance; one pair of mid-ventrolateral setae. Prothorax bearing proleg, posterior half with rectangular adhesive structure; meso- and metathoracic sternites with rectangular adhesive structure.

*Abdomen*. Sternites 1–7 modified into circular, suction cup-like adhesive structure; sternite 8 with quadrate adhesive structure; sternite 9 smooth, bearing anal proleg. Segments 1–7 lacking distinct protuberances, at most, pair of dorsolateral swellings bearing single or paired short, thin setae; single seta situated anterolaterally; lateral adhesive structure swelling bearing numerous setae, two anterolateral, two midlateral, four basalateral. Segment 8 with small dorsolateral protuberance on either side of posterior spiracular plate, each bearing pair of small setae; lateral protuberance with single seta; single short ventrolateral seta; ventral sternite bearing pair of setae. Posterior spiracular plate with sclerite encircling procerci; procercus shorter than length of spiracular plate, bearing four setae, two thick, two slender; without cone-like protuberance on either side of procerci. Terminal segment with pair of protuberances, no setae; pair of dorsolateral setae; five lateral setae; two pairs of long setae on posterior margin, above pair of anal papillae; ventral sternite lacking setae.

**Figure 20.**
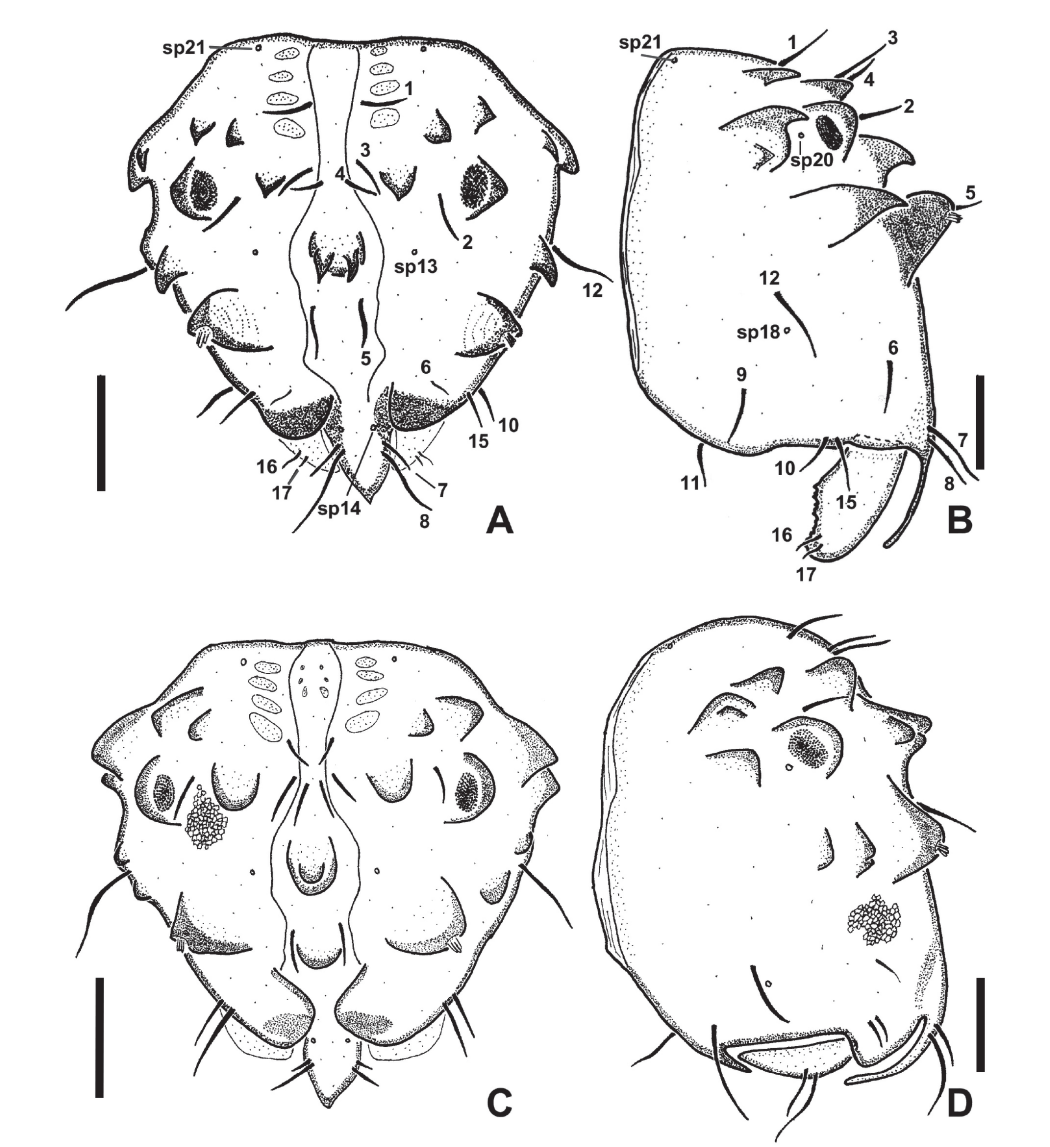
*Niphtahalteralis* group larval head capsules with setae and sensory pits numbered **A** anterior, *N.acus* sp. nov. **B** lateral, *N.acus* sp. nov. **C** anterior, *N.mapuche* sp. nov. **D** lateral, *N.mapuche* sp. nov. Abbreviations: sp, sensory pit. Scale bars: 0.1 mm.

**Figure 21.**
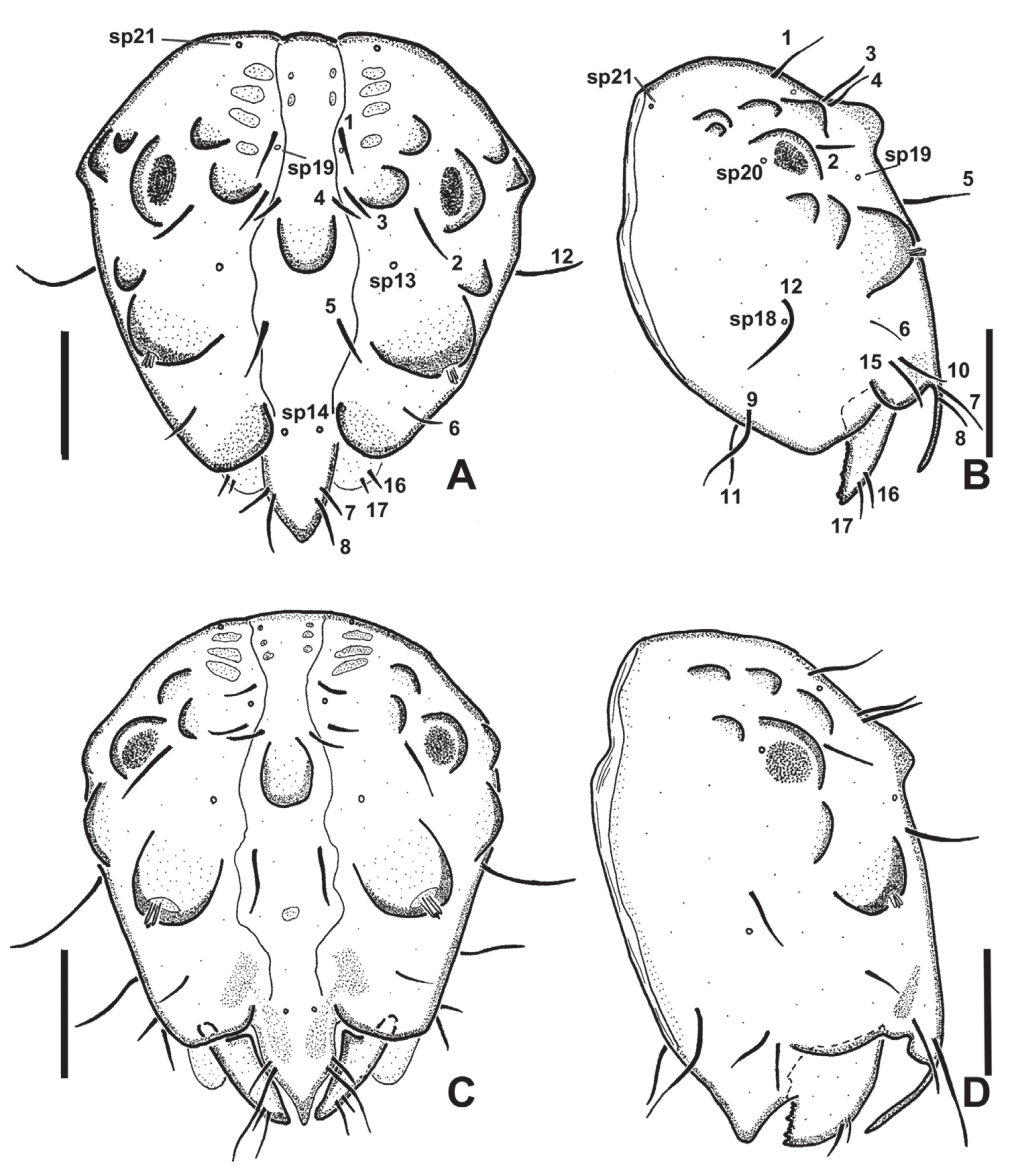
*Niphtanudipennis* group larval head capsules with setae and sensory pits numbered **A** anterior, *N.brunnea* sp. nov. **B** lateral, *N.brunnea* sp. nov. **C** anterior, *N.nudipennis***D** lateral, *N.nudipennis*. Abbreviations: sp, sensory pit. Scale bars: 0.1 mm.

#### Additional material examined.

Known only from the type series.

#### Distribution.

Known only from two localities in the Andes of southern Chile (Fig. 24B).

#### Etymology.

*Niphtabrunnea* is from the Latin *brunneus* (brown) in allusion to its brown colouration, the darkest of the *N.nudipennis* group.

#### Bionomics.

The larvae and pupae both possess the ventral adhesive structures seen in other known immature stages of South American *Niphta*. Immatures were collected from wetted vegetation in the splash zones, never from rocks (Fig. 26A–C). Vegetation included both living and dead plant material, including smooth leaves and herbaceous stems. The vegetation was not in the direct flow of water, but rather lightly splashed by water droplets that maintained enough moisture for the immatures to survive.

**Figure 22.**
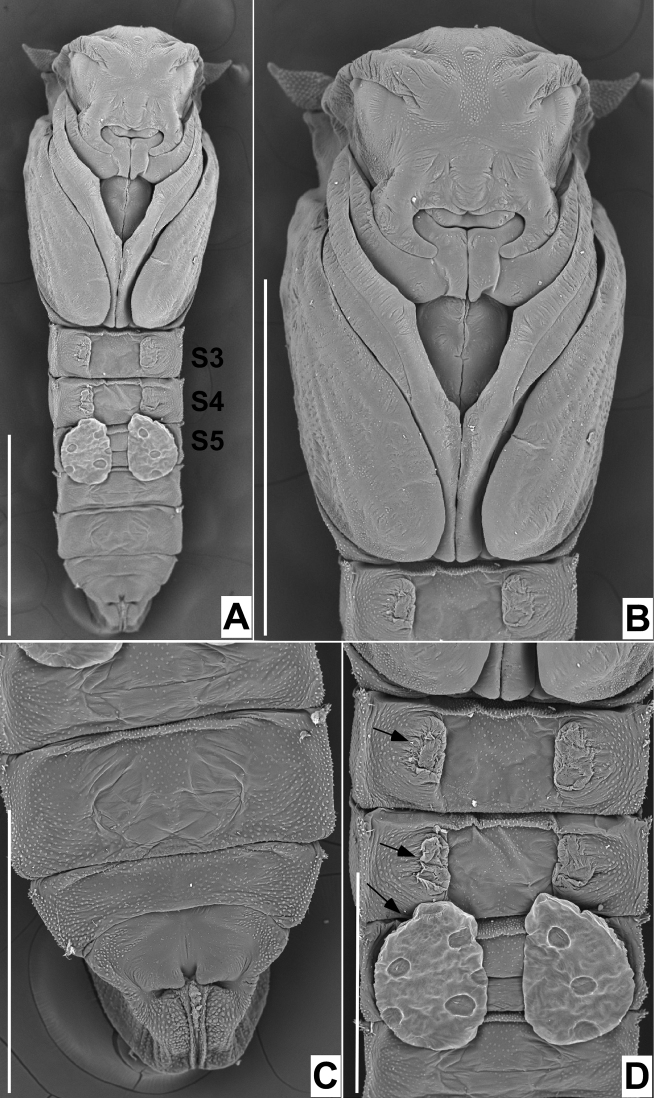
Scanning electron micrographs of ventral view of pupae of *Niphtabrunnea* sp. nov. **A** habitus (scale bar: 1.0 mm) **B** head and thorax (scale bar: 1.0 mm) **C** posterior segments (scale bar = 0.5 mm) **D** adhesive structures (scale bar: 0.5 mm). Abbreviations: S, sternite.

**Figure 23.**
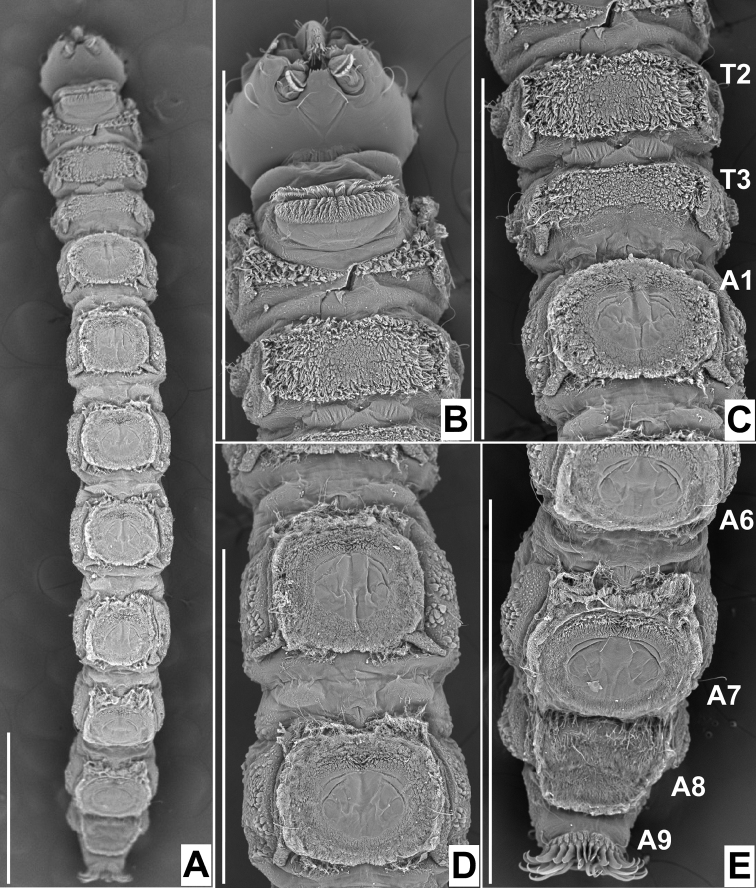
Scanning electron micrographs of ventral view of larvae of *Niphtabrunnea* sp. nov. **A** habitus **B** head and thoracic segments 1 & 2 **C** thoracic segments 2 & 3 and abdominal segment 1 **D** abdominal segments 2 & 3 **E** abdominal segments 6, 7, 8 & 9. Abbreviations: A, abdominal segment; T, thoracic segment. Scale bars: 1.0 mm.

### Niphta courtneyi

Taxon classificationAnimaliaDipteraThaumaleidae

Pivar sp. nov.

3BEF2BEC-F042-51A2-802D-7689F4AF6B53

http://zoobank.org/AE13E1F7-1030-4B4F-9492-92515EE9F67E

**Figure 24.**
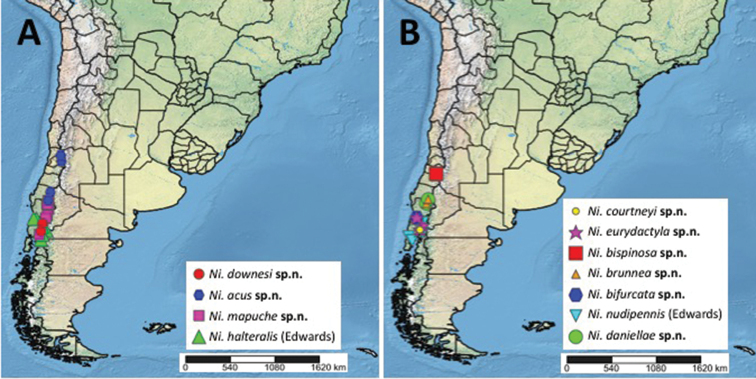
Known distribution of Chilean *Niphta***A***N.halteralis* group **B***N.nudipennis* group.

Figs 6B , 8B , 9C, H , 10C , 11B , 12B , 24B

#### Type material.

***Holotype*:** ♂, glued to point with abdomen in glycerine microvial pinned beneath, labelled: “Chile, Region X (Los Lagos)/ East side Lago Llanquihue/ small falls on road (nr PN/ VPR) 41°08.47'S 72°35.28'W/ ≈ 100 m 2.xii.2008 GW/ Courtney (CH08–30)”; “HOLOTYPE/ *Niphta*/ *courtneyi*/ Pivar [red label]” (CNC). ***Allotype***: ♀*, same data as holotype (CNC). ***Paratypes***: Chile: Region X (Los Lagos): Rte. U-99, 10.xii.2016, 41°08'28.2"S 72°35'16.8"W, elev. 101 m, roadside seeps/creek, J.K. Moulton & R.J. Pivar (1♀*).

#### Recognition.

This species is recognised by the bifurcate posterior apex of the cheliform gonostylus and the presence of three gonocoxal projections. It is lighter in colouration than the closely related *N.bifurcata*.

#### Description.

The descriptions of *N.courtneyi* differ from that of *N.bifurcata* in the following regards:

**Male.***n* = 1.

*Length* 2.1–2.3 mm.

*Colouration* (Figs 9C, 10C). Head dull, brown; pronotum and postpronotum brown; postpronotal lobe and lateral margins of prescutum yellow; scutum shiny with three distinct dark brown stripes, pleura yellow; postscutum yellow, two lateral brown spots above scutoscutellar suture; scutellum shiny, yellow; mediotergite shiny, anterior half yellow, posterior half brown; katepisternum mainly pale brown, yellow near fore coxa; remaining pteropleuron mainly yellow with dispersed brown markings; halter creamy yellow; legs pale yellow, apex of tarsi darker; abdomen brown; terminalia yellow.

*Head*. Frons with three strong setae. Flagellomere 1 expanded, 1.5 × as wide as next segment, shorter in length than 2 and 3 combined.

*Wing*. Wing length: 1.9–2.2 mm. Lightly infuscate throughout.

*Terminalia* (Figs 6B, 8B). Epandrium quadrate in ventral view, posterior margin rounded, with narrow medial cleft; long, extended beyond gonostyli; without lobes or projections. Gonocoxites oblong, longer than wide; anterior margin rounded, somewhat expanded dorsally, not closely approximated; with three spine-like projections; two anterior projections, with inner projection shorter than outer; posterior projection long, slender, tapered to single apex, 3.5 × longer than shortest projection; margin around gonostylus with long, dense thin setae. Gonostylus cheliform, dorsoventrally flattened anteriorly, swollen posteriorly; anterior apex with a few setae; posterior apex bifurcate, setose. Parameres medially fused, attached basally to arms of gonocoxal plate; divided distally into dorsal parameral apodeme and ventral arm; ventral arm projected anteroventrally toward gonocoxal plate, strongly curved anteriorly, sickle-shaped, surface textured with tiny bumps, except for smooth apex; ventral arm, when retracted, rests ventrally between dorsal arm of gonocoxal plate and inner margin of gonocoxite. Gonocoxal plate broad, well sclerotised; anterior margin subquadrate, basal margin cleft; pair of dorsal arms connected to parameres; medial aedeagal guide projected ventrally between posterior margins of gonocoxites, well sclerotised, consisting of two parts: anterior Y-shaped structure and posterior triangular plate. Cercus ovoid, only slightly visible in lateral view; projected anteroventrally; situated within epandrial indentation.

**Female.***n* = 2.

Similar to male except as follows: *Terminalia* (Figs 11B, 12B). Tergite 9 subquadrate in lateral view, 2 × as wide as tergite 8, lacking lateral projections.

#### Immature stages.

Unknown.

#### Additional material examined.

Known only from the type series.

#### Distribution.

Known only from the type locality, the East side of Lago Llanquihue in Southern Chile (Fig. 24B).

#### Etymology.

*Niphtacourtneyi* is named in honour of its collector, Gregory W. Courtney (Iowa State University). Courtney collected three new species of Thaumaleidae from Chile (*A.fredericki* Pivar, *N.courtneyi*, and *N.mapuche*), as well as immature material, prompting us to further investigate the Chilean fauna.

### Niphta daniellae

Taxon classificationAnimaliaDipteraThaumaleidae

Pivar sp. nov.

5A4DA9AB-ABB9-5166-9931-265A83DAA531

http://zoobank.org/2974B173-08B9-4832-991A-A0DD52A1929B

Figs 5A , 7A , 9D , 10D , 24B , 26A

#### Type material.

***Holotype*:** ♂, glued to point with abdomen in glycerine microvial pinned beneath, labelled: “Chile: Region IX (Araucanía)/ Rte. 71, 15.xii.2016/ 38°14'20.6"S 71°53'46.6"W/ elev. 953 m, roadside seeps/ J.K. Moulton & R.J. Pivar”; “HOLOTYPE/ *Niphta*/ *daniellae*/ Pivar [red label]” (CNC). ***Paratypes***: Chile: Region VIII (Bío Bío): Rte. Q-61, 8.xii.2016, 37°48'22.8"S 71°40'46.6"W, elev. 379 m, cascading creek, J.K. Moulton & R.J. Pivar (1♂).

**Figure 25.**
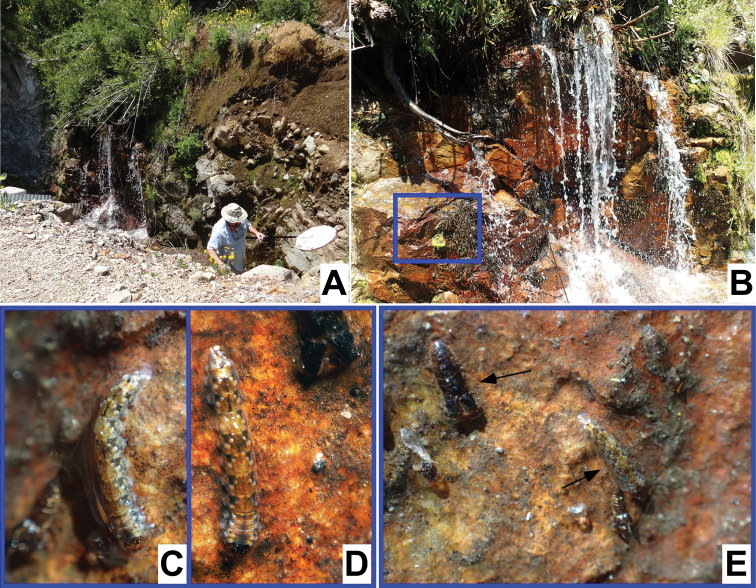
Habitat and larvae of *Niphtaacus* sp. nov. (36°55'02.7"S 71°25'49.6"W) **A** Moulton shown next to the falls for scale **B** close up of falls with box indicating where immatures were captured (Note: they were not found in high flow zones) **C** lateral view of larva, with adhesive structures visibly in contact with substrate **D** dorsal view of larva illustrating camouflage **E** larva and pupal exuviae on rock face.

#### Recognition.

This species is recognised by the sharply pointed, tapered gonostylus with no projections and not cheliform, unlike all remaining species in the *N.nudipennis* group. The gonocoxal plate also has a pair of lateral arms projected anteriorly.

#### Description.

The description of *N.daniellae* differs from that of *N.bifurcata* in the following regards:

**Male.***n* = 2.

*Length* 1.7–2.0 mm.

*Colouration* (Figs 9D, 10D). Postpronotal lobe and lateral margins of prescutum yellow; scutum shiny with three distinct brown stripes, pleura yellow; postscutum yellow, two lateral brown spots above scutoscutellar suture; scutellum shiny, yellowish; mediotergite shiny, anterior half yellow, posterior half brown; katepisternum dark brown, except yellow at base of fore coxa; anepisternum and paratergite brown; remaining pteropleuron yellow; halter entirely creamy yellow; legs yellowish brown, tarsi dark brown; abdominal tergites brown, posterior margin pale brown, sternites mainly yellow with scattered brown markings; terminalia yellowish brown.

**Figure 26.**
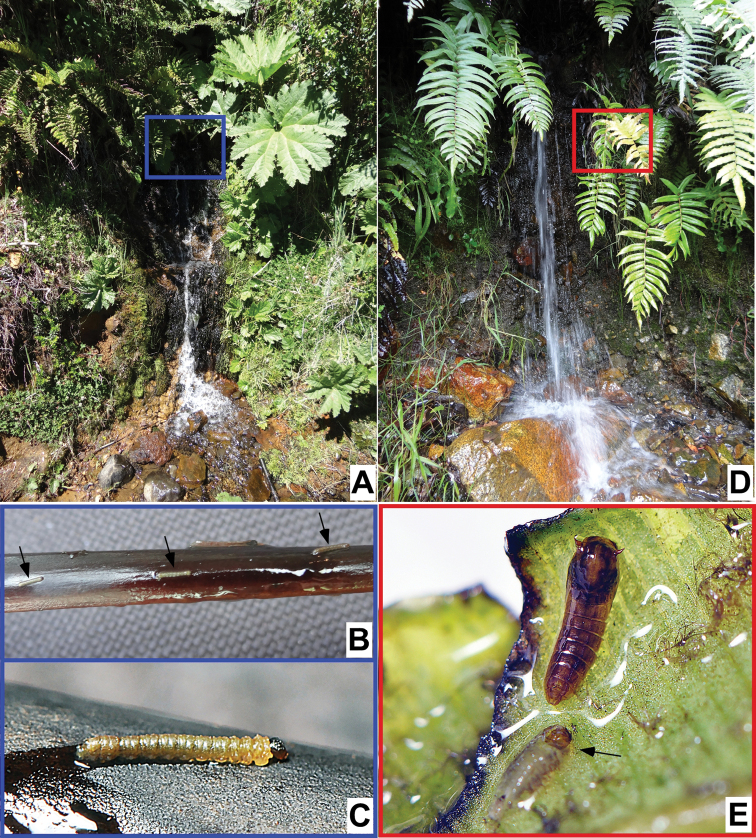
Habitat and immatures of members of the *Niphtanudipennis* group **A** type locality of *N.brunnea* sp. nov. and *N.daniellae* sp. nov., box indicating where plant stem in images **B, C** was taken from (38°14'20.6"S 71°53'46.6"W) **B** larvae of *N.brunnea* sp. nov. on plant stem from splash zone **C** close up of *N.brunnea* sp. nov. larva, adhesive structure visibly in contact with substrate **D** habitat of *N.nudipennis*, box indicating foliage in splash zone where immatures were found **E** pupa of *N.nudipennis* affixed to leaf with final instar larval exuviae visible.

*Head*. Frons with two to three strong setae. Flagellomere 1 expanded, 1.5 × as wide as next segment, subequal in length to 2 and 3 combined.

*Wing*. Wing length: 2.2–2.3 mm. Lightly infuscate throughout; bend in R_4+5_ gentle; M_4_ with slight apical bend.

*Abdomen*. Abdominal sternite 2 reduced to slender median sclerite, lacking setae; sternites 3–7 rectangular, setae restricted to posterior two-thirds; sternite 8 strongly reduced, lacking setae.

*Terminalia* (Figs 5A, 7A). Epandrium quadrate in ventral view, posterior margin rounded, with narrow medial cleft; long, extended beyond gonostyli; without lobes or projections. Gonocoxites oblong, longer than wide, inner margin setose, expanded anterodorsally above gonocoxal plate, closely approximated but not fused; two posteromedially directed spine-like projections, one anterior to gonostylus, one posterior; anterior projection pointed, bare; posterior projection blunt, setose. Gonostylus longer than wide, distal half strongly tapered to pointed apex; dorsoventrally compressed, margins curved slightly ventrally, scoopula-like, heavily sclerotised; a few setae scattered throughout, apex bare. Parameres medially fused, attached basally to arms of gonocoxal plate; surface textured with tiny bumps, except for smooth apex; divided medially into dorsal parameral apodeme and ventral arm; ventral arm projected anteroventrally, expanded medially, apical third rapidly tapered to pointed apex, sharply curved anterodorsally; ventral arm, when retracted, rests ventrally between lateral margin of gonocoxal plate and gonocoxite. Gonocoxal plate broad, well sclerotised; anteroventral margin subtriangular, basal margin cleft forming two ventrally directed projections; pair of dorsal arms connect to parameres; with median expansion projected ventrally between posterior margins of gonocoxites. Cercus ovoid, visible in lateral view; projected anteroventrally; situated within epandrial indentation.

**Female.** Unknown.

#### Immature stages.

Unknown.

#### Additional material examined.

Known only from the type series.

#### Distribution.

Known from two localities in the Andes of south-central Chile (Fig. 24B).

#### Etymology.

This species is named in honour of RJP’s wife, Danielle Lombardi, for her support during Pivar’s graduate research and entomological endeavours, and for playing an important role in organising the Chilean expedition. Raised in northern Chile (Arica), Danielle’s Spanish skills were critical for translating all communications with government and national park contacts, as well as translating our requests for collecting permits.

### Niphta eurydactyla

Taxon classificationAnimaliaDipteraThaumaleidae

Pivar sp. nov.

C274435D-5012-5285-AC62-62C1F5109C80

http://zoobank.org/3F67511B-97D8-4C71-ADF7-29936A99D333

**Figure 27.**
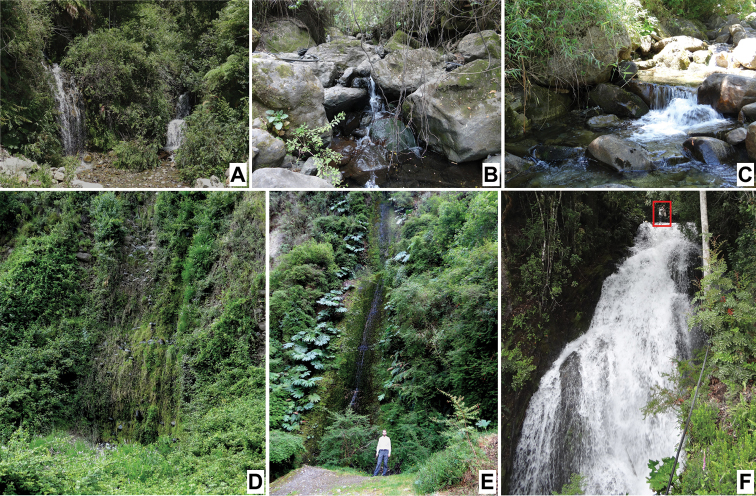
Examples of thaumaleid habitats in Chile **A** waterfalls where *Niphtadownesi* sp. nov., *N.eurydactyla* sp. nov. and *N.nudipennis* were collected by sweeping adjacent foliage (40°19'58.7"S 72°16'54.8"W) **B** collection site of *N.bispinosa* sp. nov. (34°59'48.8"S 70°48'37.0"W) (after Pivar *et al*. 2020) **C** type locality of *N.acus* sp. nov., specimens were mainly collected from plants on left (37°46'30.8"S 71°42'03.9"W) **D** rock face seep, type locality of *N.bispinosa* sp. nov. (34°59'46.7"S 70°49'19.2"W) **E** Chiloé, *N.nudipennis* was collected at a small waterfall next to the larger Cascada Tocoihue, Pivar is shown for scale (42°18'20.3"S 73°26'08.9"W); F, Puente El Salto, *N.halteralis* was collected above the falls, Pivar is shown above the falls for scale (41°31'29.2"S 72°17'14.6"W).

Figs 5B , 7B , 9E , 10E , 24B , 27A

#### Type material.

***Holotype*:** ♂, glued to point with abdomen in glycerine microvial pinned beneath, labelled: “Chile: Region X (Los Lagos)/Rte. U-99, 10.xii.2016/ 41°08'28.2"S 72°35'16.8"W/ elev. 101 m, roadside seeps/ and creek, J.K. Moulton & R.J./ Pivar”; “HOLOTYPE/ *Niphta*/ *eurydactyla*/ Pivar [red label]” (CNC). ***Paratypes***: Chile: Region X (Los Lagos): Rte. 215, 12.xii.2016, 40°40'32.4"S 72°17'35.6"W, elev. 252 m, trickle falls, J.K. Moulton & R.J. Pivar (1♂); Rte. U-99, 10.xii.2016, 41°08'09.6"S 72°35'43.3"W, elev. 81 m, roadside falls, J.K. Moulton & R.J. Pivar (1♂); Rte. U-99, 10.xii.2016, 41°08'28.2"S 72°35'16.8"W, elev. 101 m, roadside seeps/creek, J.K. Moulton & R.J. Pivar (9♂); Region XIV (Los Ríos): Antilhue, Rte. T-35, 9.xii.2016, 39°49'09.8"S 72°56'22.6"W, elev. 40 m, roadside creek, J.K. Moulton & R.J. Pivar (1♂); Rte. T-29, 14.xii.2016, 39°43'03.4"S 71°55'31.6"W, elev. 340 m, seepage, J.K. Moulton & R.J. Pivar (1♂); Rte. T-85, 13.xii.2016, 40°19'58.7"S 72°16'54.8"W, elev. 145 m, foliage around waterfalls, J.K. Moulton & R.J. Pivar (3♂).

#### Recognition.

This species is recognised by a broad, straight, finger-like projection on the gonostylus.

#### Description.

The description of *N.eurydactyla* differs from that of *N.bifurcata* in the following regards:

**Male.***n* = 17.

*Length* 1.5–1.8 mm.

*Colouration* (Figs 9E, 10E). Variable colouration, even among specimens from the same population; base colouration of thorax either yellow or orange as follows: postpronotal lobe and lateral margins of prescutum orangey/yellowish brown; scutum shiny with three distinct brown stripes, pleura yellow to yellowish brown; postscutum orangey/yellowish brown, two lateral brown spots above scutoscutellar suture; scutellum shiny, orangey/yellowish brown; mediotergite shiny, anterior half orangey/yellowish brown, posterior half brown; katepisternum mainly pale brown with scattered orange/yellow and black markings, or mostly yellowish brown with brown lateral markings above mid coxae; paratergite brown; remaining pteropleuron mainly orangey-brown to yellowish brown with dispersed markings of brown and black; base of halter creamy grey, knob pale yellow; legs pale brown, apex of tarsi darker; abdomen brown; terminalia pale brown.

*Thorax*. Antealar ridge bearing three to four setae, middle seta most pronounced.

*Wing*. Wing length: 1.8–2.2 mm.

*Abdomen*. Abdominal sternites with setae restricted to posterior two-thirds.

*Terminalia* (Figs 5B, 7B). Epandrium quadrate in ventral view, posterior margin rounded, with narrow medial cleft; long, extended beyond gonostyli; without lobes or projections. Gonocoxites oblong, longer than wide, large C-shaped scallop where gonostyli inserted; anterior margin rounded, somewhat expanded dorsally, not closely approximated; anterior inner margin with stout spine-like projection; margin around gonostylus setose. Gonostylus subquadrate; with finger-like projection emerging from posterolateral corner, gently tapered toward apex, bearing a few setae; inner anterior margin with small, rounded projection bearing a few fine setae. Parameres medially fused, attached basally to arms of gonocoxal plate; divided distally into dorsal parameral apodeme and ventral arm; ventral arm projected anteroventrally toward gonocoxal plate, strongly curved anteriorly, sickle-shaped, surface textured with tiny bumps, except for smooth apex; ventral arm, when retracted, rests ventrally between lateral margin of gonocoxal plate and gonostylus. Gonocoxal plate broad, well sclerotised; anterior margin rounded; pair of dorsal arms connect to parameres; median aedeagal guide trident-shaped, weakly sclerotised. Cercus prominent; ovoid; projected anteroventrally; situated within epandrial indentation.

**Female.** Unknown.

#### Immature stages.

Unknown.

#### Additional material examined.

Known only from the type series.

#### Distribution.

Known from both the Chilean Coastal Range and Andes of southern Chile (Fig. 24B).

#### Etymology.

The specific name is from the Greek *eury* (broad, wide) and *daktylos* (finger), in allusion to the broad, finger-like projection on the gonostylus.

### Niphta nudipennis

Taxon classificationAnimaliaDipteraThaumaleidae

(Edwards)

A1B7EFD2-5838-5CEB-B442-0F467047ADE0

Figs 1 , 5C, D , 7C, D , 9G , 10G , 11D , 12D , 14B , 15B , 16B , 17B , 18B , 19B , 21C, D , 24B , 26D, E , 27A, E

Austrothaumalea nudipennis Edwards, 1930: 113. Stuardo, 1946: 42 (catalogue); Stone, 1966: 1 (catalogue); Arnaud, 1977: 284 (distribution).
Niphta nudipennis (Edwards): Theischinger 1986: 316 (new combination); McLellan 1988: 563 (comments).

#### Type material examined.

***Holotype*:** ♂, minuten pinned with abdomen mounted in resin, labelled: “Ancud./ 17–19.xii.1926.”; “Austrothaumalea/ nudipennis Edw./ F.W. Edwards/ det. 1930.”; “S. Chile:/ Chiloe I./ F. & M. Edwards./ B.M. 1927 – 63.”; “HOLO-/ TYPE [white label with red margin]”; “NHMUK010210690”. ***Allotype***: ♀, same label data as holotype (NHMUK). ***Paratypes***: same data as holotype (4♂, 4♀, NHMUK).

#### Recognition.

This species is recognised by a long, narrow, finger-like projection on the gonostylus that has a medial bend.

#### Redescription.

The adult and immature descriptions of *N.nudipennis* differ from those of *N.bifurcata* and *N.brunnea*, respectively in the following regards:

**Male.***n* = 22.

*Length* 1.5–2.3 mm.

*Colouration* (Figs 9G, 10G). Postpronotal lobe and lateral margins of prescutum orangey-brown; scutum shiny with three distinct brown stripes, pleura orangey-brown (sometimes scutal stripes concolourous with pleura); postscutum orangey-brown, two lateral brown spots above scutoscutellar suture; scutellum shiny, yellowish brown; mediotergite shiny, anterior half yellowish brown, posterior half brown; katepisternum variable in colour, may be mostly brown with orange markings, or mostly yellowish brown with brown lateral markings above mid coxa; remaining pteropleuron mainly orangey-brown to yellowish brown with dispersed brown to light brown markings; base of halter pale yellow, turning brown, knob yellowish orange; terminalia light brown.

*Head*. Flagellomere 1 subequal in length to 2 and 3 combined.

*Wing* (Fig. 1). Wing length: 2.0–2.9 mm.

*Abdomen*. Abdominal sternite 2 with a few setae restricted to laterad on posterior third; sternites 3–7 with setae restricted to posterior two-thirds; sternite 8 lacking setae.

*Terminalia* (Figs 5C, 7C). Epandrium quadrate in ventral view, posterior margin rounded, with narrow medial cleft; long, extended beyond gonostyli; without lobes or projections. Gonocoxites oblong, wider than long, large C-shaped scallop where gonostyli inserted; anterior margin rounded, somewhat expanded dorsally, not closely approximated, extended anteriorly toward sternite 8; anterior margin with stout spine-like projection; inner margin anterior to spine setose with short, fine setulae; margin around gonostylus setose. Gonostylus quadrate basally; with pointed, finger-like projection emerging from posterolateral corner, bent 45° at midpoint, bearing a few setae at base; inner anterior margin with small, rounded projection bearing a few fine setae; posterior margin with fringe of setae. Parameres medially fused, attached basally to gonocoxal plate; divided medially into dorsal parameral apodeme and ventral arm; ventral arm projected anteroventrally toward gonocoxal plate, strongly curved anteriorly, sickle-shaped, surface textured with tiny bumps, except for smooth apex; ventral arm extends posteroventrally presumably to aid in copulation (Figs 5D, 7D); when retracted, rests ventrally between lateral margin of gonocoxal plate and gonostylus; when extended, gonostyli move inward, finger-like projections crossing and forming an ‘X’ allowing parameres to extend ventrally. Gonocoxal plate broad, well sclerotised; anterior margin rounded, basal margin cleft; pair of dorsal arms connected to parameres; with median expansion projected ventrally between gonostyli, weakly sclerotised. Cercus ovoid, slightly visible in lateral view; projected anteroventrally; situated within epandrial indentation.

**Female.***n* = 6.

Similar to male except as follows: *Abdomen*. Tergite 9 noticeably more sclerotised than preceding tergites; sternite 8 well sclerotised, with distinct blunt projection at base of hypogynial valve.

*Terminalia* (Figs 11D, 12D). Hypogynial valve not projected beyond tergite 9; posterior margin deeply cleft in ventral view, forming two subquadrate lobes; lobes densely setose, with both stout, thickened setae and thinner, long setae with slight apical bend; elongate hypogynial protuberance between valves. Tergite 9 subquadrate in lateral view, 2 × as wide as tergite 8, lacking lateral projections. Hypoproct lightly sclerotised, narrow. Cercus quadrate, projected posteroventrally; bearing numerous setae.

**Pupa.***n* = 4 (Figs 14B, 15B, 16B).

*Length* 3.0–3.1 mm.

*Colouration*. Light brown; with black spot above eyes in developing adult.

*Thorax*. 1.25 × wider than abdomen at widest point. Foreleg sheath projected straight, slightly shorter than wing sheaths; anterior half of midleg visible anterior to wing sheath, then hidden behind foreleg, apices visible, slightly longer than foreleg; hindleg concealed beneath wing sheath, only apex visible between apex of foreleg and wing sheath, longer than foreleg, extended slightly beyond wing sheath but not reaching hind margin of sternite 2. Wing sheaths not reaching posterior margin of abdominal sternite 2. Respiratory organ slightly longer than maxillary sheath, broadest subapically. Tubercle situated posterodorsally to respiratory organ, rounded, projected slightly posterolaterally; apex well separated from respiratory organ. Thorax devoid of setae.

*Abdomen*. Sternite 8 with small, indistinct lateral projection, directed slightly anterolaterally. Tergites 1–8 quadrate, devoid of setae, with pair of dorsolateral ridges (indistinct on tergites 1–6). Caudal sternite subquadrate, lacking medial lobes; posterior margin with medial longitudinal groove; without distinct caudal hooks.

**Larva.***n* = 27 (Figs 17B, 18B, 19B).

*Length of final instar* 4.7–5.4 mm.

*Colouration*. Head capsule pale brown (sometimes dark brown). Body creamy brown.

*Head capsule* (Fig. 21C, D). Five pairs of tubercles outside of ecdysial lines (not including antennal and ocular tubercle), all smaller than ocular tubercle; 2 tubercles between ecdysial lines, upper tubercle larger than lower.

*Thorax*. Spiracular protuberance bearing one pair of dorsal setae anterior to spiracle and single lateral seta.

*Abdomen*. Segments 1–7 with lateral adhesive structure inflatted bearing four setae, two midlateral, two basalateral. Terminal segment with pair of protuberances bearing pair of setae; four lateral setae, two long, two short and fine.

#### Additional material examined.

Chile: Region X (Los Lagos): Chiloé, Cascada Tocoihue, 10.xii.2016, 42°18'20.3"S 73°26'08.9"W, elev. 32 m, smaller falls, J.K. Moulton & R.J. Pivar (2♂, 4♀*); Ensenada, nr. Baños de Petrohué, 12.i.1985, J.A. Downes (13♂, 2♀, CNC); Isla Chiloé, Ancud, 12.1926, R. & E. Shannon, USNMENT01115824 (1♂, USNM) [Note: There is an additional USNM specimen identified as *N.nudipennis* from this same collection event; however, abdomen is missing and species identification cannot be confirmed (USNMENT01115825)]; Rte. V-69, 12.xii.2016, 41°19'51.5"S 72°24'40.0"W, elev. 129 m, roadside seep, J.K. Moulton & R.J. Pivar (2♂); Rte. V-69, 12.xii.2016, 41°31'48.8"S 72°17'31.2"W, elev. 39 m, trickling creek, J.K. Moulton & R.J. Pivar (5♂); same data as previous except, larvae/pupae on foliage in splash zone (27 larvae*, 4 pupae, 3 pupal exuviae); Region XIV (Los Ríos): Rte. T-85, 13.xii.2016, 40°19'58.7"S 72°16'54.8"W, elev. 145 m, foliage around waterfalls, J.K. Moulton & R.J. Pivar (1♂).

#### Distribution.

Known from both the Chilean Coastal Range and the Andes of southern Chile (Fig. 24B).

#### Bionomics.

*Niphtanudipennis* is a low-elevation species inhabiting the Valdivian temperate rainforest. Adults were collected mainly from foliage around splash zones (Figs 26D, 27A, F). The larvae possess the ventral adhesive structures found in the *N.halteralis* group; however, they were only collected from overhanging vegetation in the splash zone. Vegetation included both living and dead plant tissue, on textures spanning smooth leaf surfaces, to more textured fern fronds and herbaceous stems (Fig. 26E). Pupae were collected from the same habitats and also possess adhesive structures. The amount of water splashing on the vegetation appeared to be just enough to keep it damp enough to keep the immatures alive.

## Discussion and conclusions

### Immature stages of South American *Niphta*

Pivar et al. (2018b) described the first immatures for South American thaumaleids, where the larva and pupa of *Neothaumaleaatlantica* were described. Sinclair (2000) described the larva and pupa of *N.collessi*, an Australian species and the first for the genus. The remarkable immatures described herein are the first for South American *Niphta* and reveal unique evolutionary adaptations for the family. Unlike other described thaumaleid species, including *N.collessi*, immature South American *Niphta* are equipped with ventral adhesive structures, resembling suction cups. They are present on nearly all segments of the larva and on segments 3–5 of the pupa, and presumably aid in maintaining their position within the flow of water. The known immatures of the *N.halteralis* species group (*N.acus* and *N.mapuche*) were both collected from rocky substrates (Fig. 25), whereas the known immatures of the *N.nudipennis* species group (*N.brunnea* and *N.nudipennis*) were collected from vegetation in the splash zones, the first such observation for the family (Fig. 26).

Each group’s morphology is well adapted to their microhabitat. On a rock face, immatures have to contend with debris being washed down from the substrate above. Pupae of the *N.halteralis* group were collected from exposed microhabitats on the rock face, with no protection from flowing debris. Their stout and stocky body shape, hidden spiracles and small respiratory organs that do not extend far from the body, likely help in withstanding any potential debris impact and reduce breakage of exposed appendages. Both pupae and larvae are mottled and much darker in colouration than the pupae of the *N.nudipennis* group, offering greater camouflage from predators on the exposed rock face. Larvae have the added vestiture of dorsal tubercles, which help to break up the outline of the body. Immatures of the *N.halteralis* group also bear more setae than species of the *N.nudipennis* group, perhaps for sensing debris or predators that may be nearby.

Immatures of the *N.nudipennis* group were all collected from plant material, either living or dead, but always directly in the splash zone. They were collected from both smooth leaf surfaces and more textured vegetation, such as ferns and herbaceous plant stems. They were found on both upper and lower leaf surfaces, depending on which surface was in the splash zone. Contrary to a rock face, the amount of debris flowing down a leaf is likely minimal. Pupae of the *N.nudipennis* group are conoid, with a broad head and thorax and a narrow abdomen, protruding spiracles, and large, laterally projecting respiratory organs. All of these features are indicative of life in a habitat where debris does not pose a problem of displacement, breakage, or blockage of the respiratory organ. The leaf habitats were generally shaded and hidden from direct view, and coupled with constant splashing, individuals may experience less predation than on a rock face, thereby reducing the need for the dark, mottled colouration and tubercles. Eggs on vegetation were not observed, nor was oviposition by the adults, but they are presumably laid on the vegetation surface.

The presence of the adhesive structures is associated with behavioural traits that differ from those of other Thaumaleidae. The typical thaumaleid larva will exhibit a characteristic quick, sidewinding motion to evade predation (Thienemann 1909; Sinclair 2000; Pivar et al. 2018a). Chilean *Niphta* are much slower when trying to escape, almost caterpillar-like in movement, with slow, undulating motions. The thoracic adhesive structures are rectangular in shape and are more mobile than those of the abdominal segments, which are circular (Fig. 23).

Despite the discovery of this new larval morphotype, specimens were never collected in areas of extremely high flow or aggressive splash zones; rather, they were collected in typical thaumaleid habitat consisting of slow flowing, thin films of water. Perhaps antecedents originated in environments with higher water flow, such as in a river, and the extant species are a result of having adapted to the more familiar recent habitats. Alternatively, these slow flowing zones may be subject to torrential flooding after rainfall. The continued presence of the adhesive structures suggests a continuing evolutionary advantage in their present-day habitat.

### Comparison of *Niphta* immatures

Immatures of the Australian *N.collessi* (Sinclair 2000) differ from Chilean *Niphta* most notably in the absence of the ventral adhesive structures. Several other characters also warrant discussion.

The pupa of *N.collessi* has caudal hooks, much like in *Neothaumaleaatlantica*; however, the Chilean species lack caudal hooks or any other projections. There is also a significant reduction in setae, both in number and length. *Niphtacollessi* has numerous setae, many of which are long, while members of the *N.halteralis* group have very few, short setae. Pupae of the *N.nudipennis* group lack abdominal setae altogether. The respiratory organs of *N.collessi* are similar in appearance to those of the *N.nudipennis* group, where they are broadest subapically and project laterally, whereas those in the *N.halteralis* group are shorter and barely project laterally. The spiracles of *N.collessi* and the *N.nudipennis* group are also similar; well developed on sternites 3–7 and mounted on long, narrow, lateral tubercles. The spiracles are barely visible in the *N.halteralis* group.

The larvae also exhibit some significant differences and similarities. The most apparent difference, aside from the adhesive structures, is in the sculpture of the head capsule. All described Chilean *Niphta* have protuberances and large antennal tubercles, similar to those of *Neothaumaleaatlantica*; *Niphtacollessi* lacks protuberances and the antennae are on short tubercles. Also, much as in the pupae, Chilean *Niphta* larvae exhibit a reduction in setae compared to *N.collessi*. Another significant difference is the presence of cauliflower-like protuberances on both thoracic and abdominal tergites. These protuberances are especially prominent in the *N.halteralis* group, and are reduced but still present in *Ni.nudipennis* group. Based on Sinclair’s (2000) description, it appears that *N.collessi* lacks all protuberances and is more reminiscent of *Austrothaumalea* larvae. Similarities between the two faunas are: larval head-capsule with sensory pit 13 near dorsal margin of antenna, head-capsule only with simple setae and the caudal lobes flanking posterior spiracular plate are absent. Since descriptions of the immature stages of *N.collessi* are based on a single pupa and attached larval exuviae, additional collections of the immature stages are needed urgently to verify our comparisons.

Larvae with ventral adhesive structures have been collected in Australia. Though they have not been reared, nor identification confirmed via DNA fingerprinting (attempts to fingerprint were made, but with no success), morphology indicates these larvae are likely a member of *Niphta*. They appear very similar to members of the *N.halteralis* group: presence of distinct thoracic and abdominal protuberances, darker colouration, long abdominal setae (longer and more abundant than *N.halteralis* group) and pronounced head-capsule protuberances (not as large as *N.halteralis* group, but larger than *N.nudipennis* group). The adhesive structures appear very similar between all species: thoracic segments rectangular, adhesive structures felt-like; abdominal segments 1–7 circular, margins felt-like with smooth interior; abdominal segment 8 quadrate, felt-like (Fig. 23). Continued sampling on both continents will provide further insight into the evolution of *Niphta*.

### Faunal patterns and habitat

South American thaumaleid diversity has now increased to 17 described species. Additionally, there is the likely new species to be discovered in Ecuador, plus three more new species from Chile that were collected by the authors, but not yet described because they are represented by females only. Since males possess readily recognised genitalic features, the authors have decided to wait until further material is collected before describing these species. Both morphological and molecular data (Pivar, unpublished data) support the presence of these new species. Of the described South American species, only *Neothaumaleaatlantica* is not found in the Andes Range and is recorded from the Atlantic Forest of southeastern Brazil, in the Serra Geral mountains (Pivar et al. 2018b). The remaining species range along a roughly 2000 km stretch of the south-central Chilean Andes, with only three records from the Argentinian side, all from Bariloche. The Andes are the longest mountain range in the world at roughly 7000 km long, running from the southern tip of Chile, north to Venezuela. There are only 16 described species from ~20% of sampled mountain range. The South American fauna is undoubtedly more diverse than is presently known and current numbers are reflective of under sampling.

In Chile, thaumaleid diversity increases as one moves south. Beginning in central Chile and progressing south, the following are the known diversity of species per region (including the three undescribed females): Valparaíso (1 sp.), Santiago (2 spp.), O’Higgins (1 sp.), Maule (2 spp.), Bío Bío (5 spp.), Araucanía (7 spp.), Los Ríos (6 spp.), Los Lagos (8 spp.) and Aysén (1 sp.). These numbers are reflective of both the amount of time spent collecting in certain regions and regions sampled; some regions have likely never been sampled (particularly in northern Chile). Available habitat is also a large contributor to diversity. From Bío Bío to Los Lagos, specimens were collected in Valdivian temperate rainforests, characterised by high rainfall and cooler temperatures. Vegetation types include southern beech, laurel and broadleaved forest, bamboo and ferns. With an abundance of mountain streams and waterfalls, similar to the Nearctic Pacific Northwest (Pivar et al. 2018a), there is ample habitat for thaumaleids. From Valparaíso to Maule, habitat availability begins to drop drastically, in particular from Valparaíso to O’Higgins. These regions are part of the Chilean Matorral ecoregion of central Chile, characterised by a temperate Mediterranean climate and sclerophyllous shrubs and trees, and cacti (Meserve et al. 2019). Summers are dry and hot, and madicolous habitats scarce, as suggested by the diversity listed above. The western slopes of the Andes, from Santiago in central Chile to northern Peru, become extremely dry as they transition into the Atacama Desert. Suitable habitat is scant in these areas, though the eastern slopes of Argentina, Bolivia and Peru may contain more suitable habitat as they receive more moisture.

Most Chilean species are found in temperate rainforest regions, but some are found in both wet and dry climates (*A.chilensis* Edwards, *N.acus*). The type of madicolous habitat (i.e., creek, rock face seep, stream, waterfall, *etc.*) (Fig. 27) does not seem to dictate where a particular species may be found; oftentimes, species were collected in multiple habitat types. Multiple species and genera were frequently collected together, both within the same species group and mixed groups.

Future collections should focus on all areas of the Andes, with an emphasis on the northern sections to provide insight into the northern limits of the family and genera. Currently, the southern-most Nearctic species is *Androprosopazempoala* Sinclair and Huerta from central Mexico (Sinclair and Huerta 2010) and the northern-most South American species is the undescribed specimen from Ecuador. Thaumaleids are not recorded from Central America (Brown et al. 2009), so what is the northern limit of the South American genera? Does one genus become more abundant than the other at higher/lower latitudes or elevations? Other mountain ranges on the continent should be explored, such as the Sierras de Córdoba (central Argentina), the Sierra Nevada de Santa Marta (northern Colombia), and continued studies in Brazil will surely lead to new discoveries. Focused collecting efforts in these regions will answer these questions, as well as divulge the true breadth of South American thaumaleid diversity.

## Supplementary Material

XML Treatment for Niphta

XML Treatment for Niphta acus

XML Treatment for Niphta downesi

XML Treatment for Niphta halteralis

XML Treatment for Niphta mapuche

XML Treatment for Niphta bifurcata

XML Treatment for Niphta bispinosa

XML Treatment for Niphta brunnea

XML Treatment for Niphta courtneyi

XML Treatment for Niphta daniellae

XML Treatment for Niphta eurydactyla

XML Treatment for Niphta nudipennis

## Footnotes

see Fig. 1 for micrograph of wing with pertinent structures labelled
